# Spinal cord injury-induced neurogenic bowel: A role for host-microbiome interactions in bowel pain and dysfunction

**DOI:** 10.1016/j.ynpai.2024.100156

**Published:** 2024-04-06

**Authors:** Adam B. Willits, Leena Kader, Olivia Eller, Emily Roberts, Bailey Bye, Taylor Strope, Bret D. Freudenthal, Shahid Umar, Sree Chintapalli, Kartik Shankar, Dong Pei, Julie Christianson, Kyle M. Baumbauer, Erin E. Young

**Affiliations:** a Department of Anesthesiology, Pain and Perioperative Medicine, University of Kansas Medical Center, Kansas City, KS, United States; b Department of Cell Biology and Physiology, University of Kansas Medical Center, Kansas City, KS, United States; c Department of Cancer Biology, University of Kansas Medical Center, Kansas City, KS; d Department of Biochemistry and Molecular Biology, University of Kansas Medical Center, Kansas City, KS, United States; e Department of Surgery, University of Kansas Medical Center, Kansas City, KS, United States; f Department of Pediatrics, University of Arkansas for Medical Sciences, Little Rock, AR, United States; g Department of Pediatrics, Section of Nutrition, University of Colorado Anschutz Medical Campus, Aurora, CO, United States; h Department of Biostatistics & Data Science, University of Kansas Medical Center, Kansas City, KS, United States

**Keywords:** Spinal cord injury, Neurogenic bowel, Chronic abdominal pain, Colon dysfunction, Neurogenic inflammation, Microbiome

## Abstract

**Background and aims:**

Spinal cord injury (SCI) affects roughly 300,000 Americans with 17,000 new cases added annually. In addition to paralysis, 60% of people with SCI develop neurogenic bowel (NB), a syndrome characterized by slow colonic transit, constipation, and chronic abdominal pain. The knowledge gap surrounding NB mechanisms after SCI means that interventions are primarily symptom-focused and largely ineffective. The goal of the present studies was to identify mechanism(s) that initiate and maintain NB after SCI as a critical first step in the development of evidence-based, novel therapeutic treatment options.

**Methods:**

Following spinal contusion injury at T9, we observed alterations in bowel structure and function reflecting key clinical features of NB. We then leveraged tissue-specific whole transcriptome analyses (RNAseq) and fecal 16S rRNA amplicon sequencing in combination with histological, molecular, and functional (Ca^2+^ imaging) approaches to identify potential mechanism(s) underlying the generation of the NB phenotype.

**Results:**

In agreement with prior reports focused on SCI-induced changes in the skin, we observed a rapid and persistent increase in expression of calcitonin gene-related peptide (CGRP) expression in the colon. This is suggestive of a neurogenic inflammation-like process engaged by antidromic activity of below-level primary afferents following SCI. CGRP has been shown to disrupt colon homeostasis and negatively affect peristalsis and colon function. As predicted, contusion SCI resulted in increased colonic transit time, expansion of lymphatic nodules, colonic structural and genomic damage, and disruption of the inner, sterile intestinal mucus layer corresponding to increased CGRP expression in the colon. Gut microbiome colonization significantly shifted over 28 days leading to the increase in *Anaeroplasma,* a pathogenic, gram-negative microbe. Moreover, colon specific vagal afferents and enteric neurons were hyperresponsive after SCI to different agonists including fecal supernatants.

**Conclusions:**

Our data suggest that SCI results in overexpression of colonic CGRP which could alter colon structure and function. Neurogenic inflammatory-like processes and gut microbiome dysbiosis can also sensitize vagal afferents, providing a mechanism for visceral pain despite the loss of normal sensation post-SCI. These data may shed light on novel therapeutic interventions targeting this process to prevent NB development in patients.

## Highlights

• Mice with spinal cord injury show a similar neurogenic bowel-like phenotype to the clinical population of patients living with spinal cord injury.
• Spinal cord injury causes a neurogenic-inflammation like response in the colon.
• Alterations in microbial colonization of the gut and host gene expression may work synergistically to produce neurogenic bowel pain and dysfunction.
• Extrinsic primary afferents (spinal and vagal) may play different roles in the development of neurogenic bowel.
• Alterations in enteric and extrinsic afferent responses to chemical stimulation after SCI may serve as a cellular substrate for increased pain and dysfunction.

## 1 Introduction

Spinal cord injury (SCI) affects roughly 300,000 Americans and about 17,000 new cases arise every year (Center, 2019). Most often, these injuries are due to vehicular accidents, falls, and sports injuries. They are categorized by complete loss of sensory and motor function below the neck (i.e. tetraplegia) or partial loss (i.e. paraplegia, loss below the waist) which is dependent on the severity and location of injury to the spinal cord. In addition to loss of function, SCI comes with high personal and societal costs, including high healthcare expenses and lost wages (Center, 2019, Team, 2023). In addition to sensory and motor losses, many patients report the emergence of pain, most commonly in areas of the body below the level of injury. Neurogenic bowel (NB) is a collective term for the gut disorder affecting 60 % of SCI patients and characterized by severe abdominal pain, constipation, and bowel dysfunction (Krogh et al., 1997, Siddall et al., 2003, Stone et al., 1990, Round et al., 2021, Emmanuel, 2019, Faaborg et al., 2013, Finnerup et al., 2008, Nielsen et al., 2017). Patients most often characterize their abdominal pain as itching and/or burning and describe it as severe and relentless (Nielsen et al., 2017). NB occurs in other traumatic injuries and non-traumatic disorders of the central nervous system (i.e. traumatic brain injury, multiple sclerosis, Parkinson’s disease, etc.) (Emmanuel, 2019). However, SCI patients, in particular, list NB as one of their highest priority concerns, exceeding the loss of motor function and high healthcare costs (Finnerup et al., 2008, Burke et al., 2019, Burke et al., 2017, Hulsebosch et al., 2009, Cruz-Almeida et al., 2005, Rekand et al., 2012, Levi et al., 1995, Anderson, 2004). The few clinical studies of SCI-induced NB that are available report rapid onset gastrointestinal (GI) transit dysfunction with injured patients displaying roughly four times slower colonic transit which, combined with constipation, may further exacerbate abdominal pain (Krogh et al., 2000). Despite the rapid and persistent nature of colonic dysfunction, visceral pain has a later onset averaging 4.2 years post-injury while over a third of patients report onset after five years (Siddall et al., 2003, Finnerup et al., 2008). In contrast, other pain types (i.e. musculoskeletal and neuropathic pain) arise within 1.5 years of injury (Siddall et al., 2003). The reason for delayed visceral pain development compared to other pain types is unknown.

SCI presents a unique opportunity for NB prevention strategies. Due to the traumatic nature of most SCIs, patients usually seek immediate care, allowing treatment to be administered at the time of injury. This contrasts with conditions where NB may be common, but central nervous system damage is more gradual like multiple sclerosis and Parkinson’s Disease. However, due to the paucity of preclinical research and lack of mechanistic understanding of NB, few interventions exist for NB in any condition; the current treatments are primarily based on clinical reports and are largely symptom, rather than mechanism, focused. Laxatives and stool softeners are prescribed to treat constipation and colonic dysmotility as a first-line treatment. However, these medications have not been investigated within the context of SCI, meaning they are prescribed based on general population constipation management (Johns et al., 2021). Perhaps unexpectedly, in SCI-induced NB, these medications have low efficacy, result in unwanted side effects, and have been shown to worsen fecal incontinence (Johns et al., 2021). Most importantly, laxatives do not improve chronic abdominal pain in other conditions, such as in patients with irritable bowel syndrome-constipation (IBS-C). This suggests that GI function and abdominal pain are not necessarily linked to a single common mechanism (Vanuytsel et al., 2014), highlighting the need to explore pain-specific treatments to improve quality of life for patients with NB. Opioids, though somewhat effective for the treatment of acute pain, can exacerbate abdominal pain symptoms by increasing circular muscle contractions leading to abdominal cramps and reducing peristalsis causing slowed GI transit (Grunkemeier et al., 2007, Boland and Boland, 2017). To develop novel therapeutic strategies that protect/restore bowel function after SCI and prevent the development of SCI-induced chronic abdominal pain; there is a critical need to identify the cellular and molecular processes that are engaged following SCI.

Most of the current research on SCI focuses on recovery of function and pain reduction by targeting the site of injury in the central nervous system. Recent evidence suggests that a pathological change in both the peripheral neurons and the peripheral tissues they innervate could contribute to the development of chronic pain and organ dysfunction. Eller et al. 2022 reported findings of primary afferent hyperexcitability after thoracic SCI (Eller et al., 2022). Not only were neurons in below-level dorsal root ganglia (DRG) hyperexcitable to mechanical stimulation of the hind paw skin, they displayed increased spontaneous activity and after-discharge, two pathological changes that may underlie nociceptor-driven chronic pain (Yang et al., 2014, Walters, 2012, Bedi et al., 2010). Moreover, these changes were linked to increased protein expression of calcitonin gene-related peptide (CGRP) in below injury level hind paw skin. These data suggest that increased CGRP release in below-level tissue following SCI may contribute to changes in nociceptor sensitivity, and subsequent development of pain.

Previous work has shown that depolarization of DRG afferents by electrical stimulation medially on the axon results in terminal dumping of CGRP through antidromic activity (Kress et al., 1999). CGRP is a neurogenic inflammatory mediator, meaning that it is expressed in primary afferents and released into peripheral tissues through antidromic activity as a result of either direct neuronal damage or indirect mechanisms (i.e. vertebral bone breakage, an inflammatory environment surrounding undamaged neurons, activation of undamaged neurons through stimulation from damaged presynaptic neurons, etc.). Once overexpressed, CGRP then induces inflammation through mast cell degranulation and vasodilation leading to edema (Iyengar et al., 2017). A role for CGRP release as a mechanism underlying NB has not previously been evaluated, but the colon is dually innervated by the splanchnic and pelvic nerves that originate in thoracolumbar and lumbosacral DRGs, which are located below the level of SCI in our model and may share a common mechanism with pain originating in somatic tissues. We therefore hypothesized that, similar to the process noted in the skin, below injury level primary afferents could be expressing and releasing CGRP in the colon through antidromic activity thereby disrupting colon function and propagating visceral pain.

There is a paucity of preclinical studies into the mechanisms of SCI-induced NB development, so we aimed to: 1) characterize early-onset phenotypic changes that occur within the bowel and in the specific subpopulation of peripheral neurons innervating the bowel and 2) examine correlations between early-onset phenotypes and long-term consequences that could point to novel preventative and/or treatment targets for NB. Consequently, we hypothesize that injured spinal afferents release CGRP into below-level, innervated tissue. Specific to NB, this neurogenic inflammation-like process appears to damage colon structure and impair function. Moreover, this process precedes gut dysbiosis which we show sensitizes the colon specific neurons providing a feed-forward mechanism for chronic abdominal pain development and perpetuation.

## 2 Methods

### 2.1 Mice

Adult (8–10 week old) female C57BL/6J mice from Jackson Labs (Bar Harbor, ME) were housed at the University of Connecticut Health Center (UCHC) or the University of Kansas Medical Center in animal facilities on a 12:12 light: dark cycle with food and water available *ad libitum* and a fiber nestlet available. Animal use protocols conformed to NIH guidelines and were approved by the Institutional Animal Care and Use Committees at the University of Connecticut Health Center and the University of Kansas Medical Center and conformed to the Committee for Research and Ethical Issues of IASP.

### 2.2 Spinal contusion injury

Mice were anesthetized with isoflurane (5 % induction, 2 % maintenance) and a laminectomy was performed at thoracic 9 (T9) and the Infinite Horizons Impactor (65 kDynes of force, one second dwell time) was used to deliver a moderate severity contusion injury (Cheriyan et al., 2014). Injured mice were placed in a new cage (Tecniplast with cobb bottom bedding) on top of a heating pad until ambulatory and then received daily 5 mg/kg of gentamicin sulfate via intraperitoneal (i.p.) injections for five days to prevent post-surgical infection (Bhalala et al., 2013). In addition, investigators manually voided mouse bladders using the Credé maneuver twice daily until normal function was regained. SCI rodents were assessed one day (D1), seven days (D7), or 28 days (D28) after injury, and naïve, uninjured mice were used as a control group for all experiments. Prior to project initiation, we established that any mice showing autotomy, weight loss greater than 20 % of their starting body weight, or signs of respiratory distress would be euthanized and excluded from the study. No mice met these criteria. Because SCI is a polytraumatic injury that can include damage to multiple tissues, including skin, muscle, and bone, that collectively influence pain development, and because prior work has shown that performing laminectomies alone in sham controls is sufficient to produce nociceptor sensitization and long-lasting pain in mice (Odem et al., 2018, Odem et al., 2019), we used naïve mice as controls to understand how pathophysiological conditions produced by polytraumatic SCI may affect colon structure and/or function compared to the uninjured, naïve state. While useful for showing the effects of polytraumatic SCI, this experimental design does limit experimental inferences that can be made about the effects of SCI alone. All studies were approved by the University of Connecticut or University of Kansas Medical Center Institutional Animal Care and Use Committee and treated in accordance with published NIH standards.

### 2.3 Basso mouse Scale

The Basso Mouse Scale (BMS) for locomotion defects was conducted as previously described (Basso et al., 2006). Mice with an SCI were individually placed on a flat surface and allowed to freely move for four minutes. Motor function was assessed using the scale for each hind leg, and the left and right hind leg scores were averaged for the overall mouse score.

### 2.4 Colonic motility

Colonic transit was measured using a bead assay as previously described (Vilz et al., 2012). Mice were anesthetized using isoflurane (5 % induction, 2 % maintenance) and a 2 mm diameter glass bead (Sigma-Aldrich) was gently pushed intrarectally 3 cm into the distal colon using a fistula probe (Roboz Surgical Instrument Co., Inc.). A stopwatch was immediately started, and colonic transit time was calculated based on latency to bead expulsion.

### 2.5 Gastrointestinal motility

Gastrointestinal motility was measured using high molecular weight (70,000 kDa) Fluorescein isothiocyanate–dextran (FITC-Dextran) (Sigma-Aldrich) as previously described (Vilz et al., 2012). Mice were anesthetized with isoflurane (5 % induction) and a 22-gauge gavage needle was used to orally deliver 100 μL of FITC-dextran. Mice were returned to their home cages for 90 min at which point they were sacrificed using an overdose of isoflurane and decapitation. The gastrointestinal tract from stomach to rectum was removed from the mouse and cut into equal length segments. The stomach and cecum were maintained as intact segments, the small intestine was separated into ten equal segments, and the large intestine was separated into three equal segments. Each segment was flushed with 1 mL of Krebs-Henseleit Buffer into 2 mL microcentrifuge tubes and spun down for 10 min at 12,000rcm. The supernatant was collected in a new microcentrifuge tube prior to sample duplicates being loaded onto a 96-well plate and read at 492 nM using a SmartReader 96 Microplate Absorbance Reader (Accuris Instruments). The geometric center of fluorescence in the gastrointestinal tract was calculated using the following formula (Vilz et al., 2012):

GC = ∑ (% of total fluorescent signal per segment * segment number) / 100

### 2.6 Fecal water weight assessment

Four fresh fecal pellets from each mouse were weighed in grams (g) to four decimal places. The pellets were then microwaved for six minutes at power level four in a microwave (60 Hz 1500 W 2450 MHz). Following dehydration, the pellets were weighed again. The wet weight and dry weight were normalized by dividing each weight by the number of pellets. The difference between the normalized wet and dry weights represents the water content (g) and was used in all statistical analyses.

### 2.7 Whole transcriptome RNA sequencing

Naïve and SCI mice at 1 or 7 days after injury were euthanized with an overdose of isoflurane prior to intracardiac perfusion with ice cold Ca^2+^/Mg^2+^-free Hank’s Balanced Salt Solution (HBSS, Invitrogen), and then a 2.5 cm segment of the distal colon was harvested. Tissue samples were flash frozen on dry ice and stored at −80 °C for batch processing. Total RNA was extracted using RNeasy isolation columns (Qiagen, Hilden, Germany) and all samples had a 260/280 ratio of 1.85–2.1. Whole transcriptome RNA sequencing was then conducted on the colon samples using the Illumina platform to determine transcript abundance.

Library validation was performed using the DNA 1000 ScreenTape (Agilent Technologies) on the TapeStation 4200. The concentration of each library was determined by qPCR using a Roche Lightcycler96 using FastStart Essential DNA Green Master (Roche 06402712001) and KAPA Library Quant (Illumina) DNA Standards 1–6 (KAPA Biosystems KK4903). Libraries were pooled based on equal molar amounts to 1.9 nM for multiplexed sequencing. Pooled libraries were denatured with 0.2 N NaOH (0.04 N final concentration) and neutralized with 400 mM Tris-HCl pH 8.0. A dilution of the pooled libraries to 380 pM was performed, followed by onboard clonal clustering of the patterned flow cell using the NovaSeq 6000 S1 Reagent Kit v1.5 (200 cycle; Illumina). A 2x101 cycle sequencing profile with dual index reads was completed using the following sequence profile: Read 1 – 101 cycles x Index Read 1 – 8 cycles x Index Read 2 – 8 cycles x Read 2 – 101 cycles. Following collection, sequence data was converted from.bcl file format to fastq file format using bcl2fastq software and de-multiplexed into individual sequences for data distribution using Illumina BaseSpace.

For further downstream analysis, the raw sequence data was QC checked by FastQC tool (http://www.bioinformatics.babraham.ac.uk/projects/fastqc). To generate gene counts, we aligned the samples to mouse genome by using the RSEM [PMID: 21816040]. Sequencing quality and alignment statistics were inspected before downstream statistical analysis. Pair-wise comparisons were performed using the Bioconductor package “edgeR” [PMID: 19910308]. Data were normalized by library size and low/non-expressed genes (<1 count per million (CPM) in ^3^2 samples) were filtered from analysis, resulting in 17,793 genes being retained for pair-wise analysis. the Bioconductor package “edgeR” (PMID: 19910308) was used to identify differentially expressed genes with an FDR ≤ 0.05 to identify transcripts based on significant upregulation or downregulation. Gene ontology (GO) term analysis was performed using Metascape (Zhou et al., 2019).

We then produced prioritized gene sets based on a common differential regulation pattern following SCI (compared to naïve). Genes identified in these prioritized lists at both days 1 and 7 were extracted and sorted into two categories based on common differences in direction of regulation: day 1 and day 7 were both upregulated or day 1 and day 7 were both downregulated. These lists of transcripts were then further divided into three sub-categories or trajectories by comparing the differential expression at day 7 to that present at day 1: higher gene expression at day 7, lower gene expression at day 7, or no change between day 1 and day 7. No change was defined as gene expression at day 1 and 7 that fell within one standard deviation of the median of the FDR. Greater or lower than gene expression was defined as gene expression that exceeded one standard deviation above or below the median of the FDR. Venn diagrams of upregulated and downregulated genes were developed using JVenn (Bardou et al., 2014). To identify the transcript expression signature for each trajectory, we used Over-Representation Analysis (ORA) using Webgestalt (Liao et al., 2019) to compare the list of transcripts within each trajectory category and searched for all enriched gene ontology (GO) terms by trajectory. These GO terms were sorted into broad categories based on common functional groupings were further evaluated with a literature search for their prevalence in “pain”, “visceral pain”, and “hypersensitivity” in PubMed indexed publications and links between categories that were over or under-expressed.

### 2.8 Single cell gel electrophoresis comet assay

Double-stranded DNA breaks from colon samples were quantified using the OxiSelect^TM^ Comet Assay Kit (3 Well Slides), also known as single-cell gel electrophoresis (Cell BioLabs, Inc) according to manufacturer’s instructions. Tissue samples were minced with a razor and further homogenized using a mortar and pestle. In order to generate a positive control, naïve tissue was minced and soaked in 0.1 % hydrogen peroxide for 20 mins and then resuspended in ice-cold PBS. DNA “comets” were visualized on a Nikon 80i epi-fluorescent microscope and images captured at 10X magnification. 20 DNA comets were analyzed per mouse. The images were blinded, and two independent experimenters rated all comet tails on a scale from one to five with one representing little to no comet tail and five representing a full tail, as previously described (Kumaravel et al., 2009). Experimenter scores were averaged for all comets and this average used for all subsequent analyses.

### 2.9 Histology and immunohistochemistry (IHC)

Roughly 4.5 cm of the distal colon was post-fixed in 10 % neutralized formalin. After 24 h, the fixed colons were washed with 70 % ethanol, embedded in paraffin wax, and cut into 8 μm thick cross-sections using a microtome and were mounted on glass slides. After dewaxing and rehydrating the tissue using a ClearRite-ethanol gradient, slides were stained for goblet cells and mucins using an Alcian Blue dye and Nuclear Red Fast counterstain kit (Abcam). Quantification of percent area of Alcian blue staining was calculated using the Alcian Blue color deconvolution plugin in FIJI (Schindelin et al., 2012). A terminal deoxynucleotidyl transferase dUTP nick end labeling (TUNEL) assay stain was conducted using a TUNEL kit with HRP-DAB substrate (Abcam). IHC was conducted using standard techniques (Aviva Systems Biology). A rabbit anti-CGRP primary antibody (Peninsula Laboratories International, Inc.), a chicken anti-PGP9.5 neuronal marker antibody (Invitrogen), a goat anti-rabbit Alexa Fluor 488 secondary antibody (Life Technologies), and a goat anti-chicken Alexa Fluor 555 secondary antibody (Life Technologies) was used for IHC. Slides were counterstained with Vector Mounting Medium with DAPI (VectaShield). All images were taken using a Nikon 80i microscope.

### 2.10 Fluorescent *in situ* hybridization

Fluorescent *in situ* hybridization (FISH) for localization of bacteria in the intestinal lumen was conducted as previously described (Ahmed et al., 2018). Mice were sacrificed at their respective timepoints using isoflurane gas overdose followed by intracardiac perfusion of ice-cold Hanks Balanced Salt Solution. About 4.5 cm of the distal colon was collected and post-fixed in MethaCarnoy’s fixative. After fixation, tissue was embedded in paraffin wax and cut in 8 μm thick cross-sections using a microtome and mounted on glass slides. After dewaxing and rehydrating tissue using a ClearRite-ethanol gradient, slides were stained using 5 μg/ml of EUB338 probe. EUB338 is a DNA oligo probe designed to be complimentary with a generalized bacteria sequence (5′-GCTGCCTCC CGTAGGAGT-3′), and this probe was conjugated with a TEX615 fluorophore (Integrated DNA Technologies) for fluorescent microscopy (Nikon i80 microscope) visualization of microbe localization. Slides were counterstained with Vector Mounting Medium with DAPI (VectaShield).

### 2.11 Microbiome sequencing

Stool samples were collected and immediately flash frozen in phosphate buffered saline for storage until batch processing. Bacterial rRNA was extracted and purified using a QIAamp Fast DNA Stool Mini Kit (Qiagen) according to manufacturer’s instructions. Samples were normalized to 5 ng/μL of total DNA for processing by the Genome Sequencing Core in the Center for Molecular Analysis of Disease Pathways at KU-Lawrence. Sequencing libraries were constructed using Amplicon PCR of V3/V4 16S with overhang adapters attached, followed by cleanup of the Amplicon PCR to purify the amplicons away from free primers, Index PCR to attach dual indices and Illumina sequencing adapters using the Nextera XT Index Kit (Illumina) and cleanup of the Index PCR to generate the final sequencing library.

After quantification of the sequencing libraries with Qubit, the sequencing libraries were pooled together equally by ng amount, and the nM concentration verified with an Illumina KAPA Library Quantification qPCR assay (Roche). An Illumina MiSeq was used to generate paired-end, 250 base pair sequence reads from the libraries in a multiplexed fashion. The raw sequences were analyzed using the DADA2 pipeline in Quantitative Insights Into Microbial Ecology (QIIME2). All steps to preprocess, trim, merge and perform denoising and sequencing alignment were conducted as in previously established methods (Brink et al., 2020, Wankhade et al., 2019, Brink et al., 2019, Mercer et al., 2020).

### 2.12 Retrograde labeling of colon specific, extrinsic primary afferents

Retrograde labeling was carried out seven days before sacrifice for tissue collection. Mice were anesthetized with gaseous isoflurane (5 % induction and 2 % maintenance), and a laparotomy was conducted to open the abdominal cavity. Each mouse received a series of injections of Alexa Fluor 488-conjugated cholera toxin B (CTB) (Invitrogen) at a concentration of 1 mg/mL. These injections were inserted into the subserosa of the distal colon using a 1-inch, 33-gauge Hamilton needle with an angle of 12 and point style of 4 (Hamilton Company). The cumulation of the injections’ total volume was 10 μL per mouse, and then the abdominal wall and skin were sutured, and mice were individually housed for recovery.

### 2.13 Calcium imaging

Ca^2+^ imaging was conducted as previously described (Malin et al., 2011, Christianson et al., 2010). Mice were sacrificed using isoflurane gas overdose followed by intracardiac perfusion of ice-cold Hanks Balanced Salt Solution. Bi-lateral nodose ganglion (ND) (Pierce et al., 2015) and bi-lateral DRG separated by thoracolumbar and lumbosacral segments (T11-L2 and L3-S1, respectively) were collected and dissociated (Haring et al., 2020). In addition, the myenteric plexus, representing approximately 80 % of the total neurons of the enteric nervous system, was removed from the intestinal wall and dissociated as previously described (Malin et al., 2007). All neuronal subpopulations were plated onto 12-mm poly-D-Lysine/laminin coverslips (Corning Inc.). 16–18 h after dissociation, all cells were incubated in 1.5 mL of an incubation solution (containing 3 μL of FURA-2 AM (Invitrogen) and a concentration of 5 mg/mL BSA (Sigma-Aldrich) in 1x HBSS) at 37˚C for 30 min.

Following FURA incubation, the coverslips were mounted on an inverted Nikon TiE microscope stage (Nikon) with a Photometric HQ2 dual-mode cooled CCD camera (Boyce Scientific). 1x HBSS constantly flowed over the coverslip at 5 mL/min, controlled by a gravity flow system (Warner Instruments). Cells were treated with agonists through a gravity-feed pinch valve control perfusion system (Warner Instruments). The cells were excited by a 175 W Xenon illumination system with high-speed filter wheel and responses were measured as the ratio of 340/380 nM excitation and 510 nM emission (ΔF_340/380_). Cells were briefly (5 sec) exposed to 30 mM K^+^ solution (High K^+^). Response to High K + was used as criteria for identification of cells as “living” with a peak Ca^2+^ influx (ΔF) cutoff of at least 0.1. Five minutes after High K+, cells were then briefly stimulated with 1 μM capsaicin followed by fecal supernatants (10 min after capsaicin) to determine cellular responses to these agonists.

Fecal supernatants were created by removing three pellets of fresh feces from a rodent at the time of sacrifice and stored at −20˚C until use (no more than three months). The morning of calcium imaging, the three fecal pellets were homogenized in 1 mL of colorless 1x HBSS, centrifuged for 30 s to pellet the fecal material, and the remaining supernatant was diluted 1:1 with colorless 1x HBSS for a final working solution. During imaging, 75 μL of the fecal supernatant was carefully pipetted onto the coverslip. In post-imaging analysis, cells were sorted by presence of CTB (i.e. colon specific), and the peak Ca^2+^ influx (ΔF) and proportion of cells that responded to each agonist were calculated.

### 2.14 Statistical analyses

All statistics were calculated using Statistical Package for the Social Sciences (SPSSvs27, IBM). All data are presented as mean ± standard error of the mean except for Ca^2+^ figures where the proportion of cells are presented. A univariate analysis of variance (ANOVA) was conducted followed by the LSD post-hoc test for most assays. To assess the ΔF in Ca^2+^ imaging, we conducted a two-way ANOVA for overall effects followed by individual one-way ANOVAs and LSD post-hoc tests within each cell population (i.e. CTB + or unlabeled). To assess the proportion of cell response in Ca^2+^ imaging, a χ^2^-squared test was used within each population. If there was an overall significance, we conducted follow up χ^2^-squared post hoc tests for each timepoint compared to naïve. The ΔF for heterologous gut lumen content Ca^2+^ imaging was evaluated using a two-way ANOVA for overall effects followed by independent samples *t-*tests between conditions within each cell population. A significance level of *p ≤* 0.05, denoted by the * symbol, was applied to all analyses.

## 3 Results

### 3.1 T9 SCI induces NB phenotypes in mice as reported in clinical populations

**Fig. 1:**
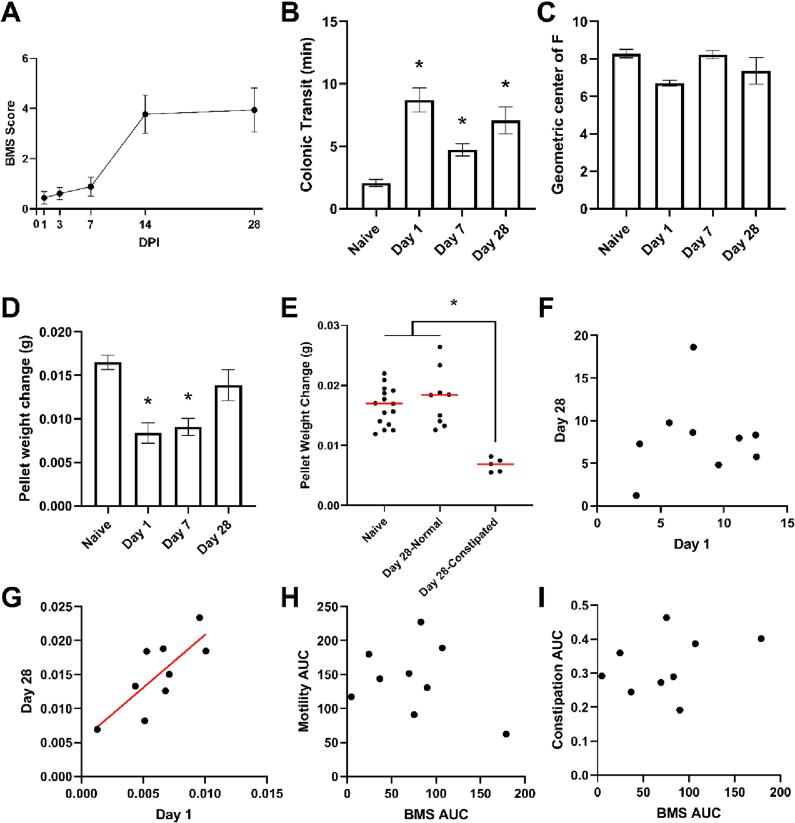
T9 SCI induces gut motility dysfunction and constipation in mice, which correlates with human symptoms. **(A)** Basso Mouse Scale (BMS) for locomotion showed sufficient inhibition of locomotor function as a validation of successful SCI. There is an increase in BMS score through D28 which is reflective of recovery. **(B)** The mice show significant differences in gut motility function at all timepoints after injury compared to naïve, n = 14 per condition, *p < 0.05. **(C)** There is no significant difference in whole gastrointestinal transit time, n = 14 per condition. **(D)** Constipation was measured as a change in pellet weight before and after microwave dehydration. There is a significant difference for D1 and D7 compared to naïve, however D28 was not significantly different, n = 14 per condition, *p < 0.05. **(E)** D28 mice in the constipation assay have diverging phenotypes whereas some mice retain constipation (n = 5) and others recover stool consistency (n = 9). D28 mice were separated by constipation scores into those that rated below the lower range of naïve mice (constipated group) and those within the range of naïve mice (normal group). The D28- constipation group was significantly different compared to both naïve and the D28- normal group. The red line represents the mean, *p < 0.05. **(F,G)** Repeated measures of colonic transit time (CTT; **F**) and constipation (**G**) were taken at D1 and D28 after SCI. For CTT, the Pearson correlation coefficient was 0.10 (*p =* 0.79) and the Pearson correlation coefficient for constipation was 0.077 (*p =* 0.01), n = 9. There was no significant association between early colonic transit defects, but early constipation was significantly associated with chronic constipation. The red line represents the line of best fit. **(H,I)** Repeated measurements of BMS, colonic transit, and constipation were collected for each mouse over the time-span of 28 days. The area under the curve was calculated for individual mice per outcome. Correlation for BMS versus CTT (**H**) and BMS versus constipation (**I**) were calculated using a Pearson correlation. There was no significant correlation between BMS and either outcome. The Pearson correlation coefficient for BMS versus colonic motility was −0.32 (*p =* 0.41), and the Pearson correlation coefficient for BMS versus constipation was 0.33 (*p =* 0.39). N = 9. (For interpretation of the references to color in this figure legend, the reader is referred to the web version of this article.)

Using the Basso Mouse Scale (BMS) (Basso et al., 2006) as a measurement of locomotor recovery, we confirmed efficient locomotor impairment as expected following moderate severity contusion SCI (Fig. 1A). Colonic transit time (CTT; glass bead assay) was significantly different post-SCI with a one-way ANOVA confirming a significant main effect of condition (Fig. 1B; F(3,53) = 14.680, *p =* 0.000). Post hoc comparisons revealed that the effect of condition was due to significant differences between naïve mice and all times post-SCI (D1 (*p =* 0.000), D7 (*p =* 0.016), D28 (*p =* 0.000)). We next quantified total gastrointestinal (GI) motility by measuring the geometric center of fluorescent intensity of FITC-dextran (Fig. 1C). There was no significant effect of condition (F(3,21) = 1.288, *p =* 0.305), suggesting a colon specific motility defect versus disruption across the entire GI tract.

Constipation was assessed by measuring water weight loss after stool dehydration (in grams/pellet). There was a significant main effect of condition (Fig. 1D; F(3,53) = 10.124, *p =* 0.000) with a post hoc analysis revealing a significant difference between naïve and D1 (*p =* 0.000) and D7 post-SCI (*p =* 0.000), but there was no significant difference between naïve and D28 post-SCI (*p =* 0.133). However, upon further analysis, we identified two diverging phenotypes at D28 post-SCI, with 35.7 % of the mice measuring water weight loss below the lowest range of naïve (i.e. constipated) versus the remaining mice showing normal stool consistency within the range of the naïve condition (Fig. 1E). There was a significant main effect of condition (F(2,26) = 17.923, *p =* 0.000) where the D28-constipated group was significantly different than both naïve (*p =* 0.000) and the D28-normal group (*p =* 0.000). A prior clinical report (Siddall et al., 2003) suggests that early onset pain occurrence may be a predictor for more severe chronic pain following SCI. Along these lines, we assessed whether early onset NB phenotypes were also an early predictor of severe, chronic NB by linear associations between symptoms at D1 and D28 in the same mice. While CTT at D1 and D28 were not significantly correlated (Fig. 1F; r*_p =_* 0.10, *p =* 0.79), constipation at D1 and D28 were found to have a strong positive correlation (Fig. 1G; r*_p =_* 0.077, *p =* 0.01). While motility defects do not appear to be early predictors of subsequent NB symptom burden, constipation did show a significant relationship with more severe chronic symptoms.

Prior reports suggest a potential association between locomotor recovery and NB symptom improvement (Zhang and Hu, 2013), so we conducted a series of analyses as a preclinical investigation of these relationships. We calculated the area under the curve for BMS, colonic motility, and constipation for each mouse and computed a Pearson correlation coefficient (r*_p_*) to assess the linear relationship between BMS and the NB phenotypes. BMS was not significantly correlated with colonic motility (Fig. 1H; r*_p =_* −0.32, *p =* 0.41) or severity of constipation (Fig. 1I; r*_p =_* 0.33, *p =* 0.39) suggesting that NB severity is largely independent of locomotor dysfunction/recovery and should be assessed as a separate disorder post-SCI.

### 3.2 RNA sequencing suggests genomic instability and neurogenic inflammation in the distal colon after SCI

**Fig. 2:**
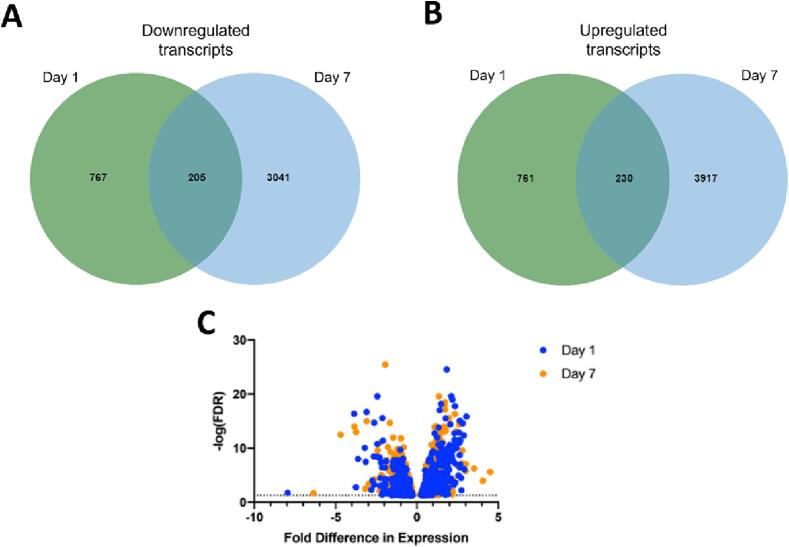
8,921 mRNA transcripts were differentially expressed in the colon following SCI. **(A,B)** Venn Diagrams of downregulated **(A)** and upregulated **(B)** transcripts at day 1 and day 7 after SCI compared to naïve. The overlapping portion of each diagram represents the number of genes that are differentially expressed at both timepoints. **(C)**Volcano plot displays the filtered gene sets and their overall direction.

Recent work from our lab and others, point to early engagement of peripheral mechanisms in the development of pain and dysfunction after SCI (Eller et al., 2022), though NB has not been assessed explicitly. In line with this prior work, RNA sequencing (RNAseq) was conducted on the distal colon at both D1 and D7 post-SCI to identify high priority candidate genes and biological processes involved in initiation of NB early after SCI. Colon samples from three mice per condition were pooled and used to perform multiple comparisons between groups. FastQC results indicated all samples successfully passed quality control. Pairwise comparisons were conducted separately to compare naïve mice to D1 and D7 post-SCI, and a list of differentially expressed genes were generated based on an FDR ≤ 0.05. A total of 8,921 genes were significantly upregulated or downregulated in the colon following SCI compared to naïve at a minimum of one time point. Of these genes, 435 were found to be differentially expressed at both D1 and D7, reflecting a common set of gene transcripts with sustained alterations in expression across the first week post-SCI (Fig. 2).

The prioritized gene sets identified for D1 and D7 post-SCI were then further categorized based on the direction of differential expression (i.e. upregulation or downregulation). Subsequent analyses focused entirely on prioritized transcripts where the pattern of differential expression was in the same direction (i.e. where both D1 and D7 were upregulated or downregulated compared to naïve). This yielded 205 downregulated and 230 upregulated genes and in common between D1 and D7.

**Fig. 3:**
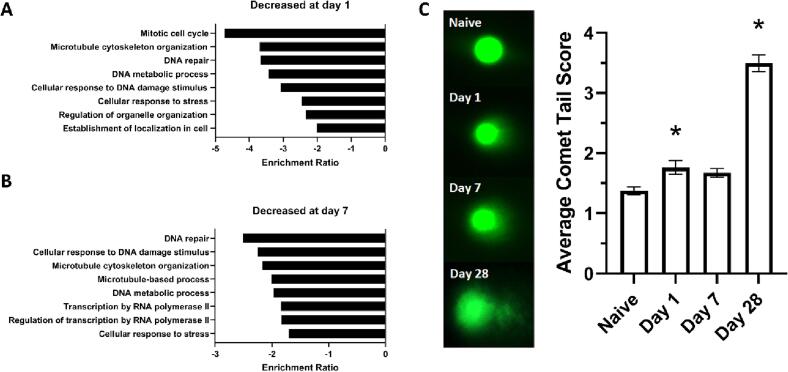
DNA repair mechanisms have reduced expression in the colon after SCI with an associated accumulation of double stranded DNA damage breaks. **(A, B)** Gene Ontology terms for downregulated RNA transcripts in the colon D1 **(A)** and D7 **(B)** after SCI. **(C)** Single cell gel electrophoresis comet assay quantification of double stranded DNA breaks in the colon after injury. The images on the left are representative comets from each condition. Quantification of average comet tail score is depicted in the graph on the right. There was a significant increase in DNA damage for both D1 (n = 4 mice with 20 cells per mouse, *p =* 0.027) and D28 (n = 5 mice with 20 cells per mouse, *p =* 0.000) compared to naïve (n = 60). D7 was not significantly different using a post-hoc test but trending towards significance (n = 5 mice with 20 cells per mouse, *p =* 0.072). Bar graph is depicted as mean ± (SEM). One way ANOVA, LSD post-hoc, *p < 0.05.

Analysis of GO terms enriched with downregulated genes showed reduced expression of DNA repair mechanisms at both D1 and D7 individually (Fig. 3A, B) with specific overlapping genes listed (Supp. Table 1, Table 2). Single Cell Gel Agarose Comet Assay was used to validate this downregulation of DNA repair by quantifying accumulation of genomic DNA damage in the colon 1, 7, and 28 days after SCI (Fig. 3C). ANOVA revealed a significant main effect of condition (F(3,336) = 80.576, *p =* 0.000). A post hoc analysis (Fisher’s LSD) confirmed that this effect was driven by significant differences between naïve and D1 (*p =* 0.027) as well as naïve and D28 conditions (*p =* 0.000), such that significantly more DNA damage accumulation was observed after SCI. D7 was not significant compared to naïve but trending towards significance (*p* = 0.072).

**Table 1:** Genes downstream of CGRP are significantly increased in the colon seven days after SCI compared to naive. FDR and logFC were obtained from RNA sequencing.

**Gene**	**Encoded protein**	**FDR**	**logFC**
*Ramp1*	Receptor Activity Modifying Protein 1 (RAMP1)	0.0022	0.9754
*Gnas*	G-Protein Alpha Subunit	0.0072	0.3158
*Prkaca*	Protein Kinase A Catalytic Subunit Alpha	0.0158	0.3102
*Mapk1*	Mitogen-Activated Protein Kinase 1 (ERK)	0.0072	0.3353
*Creb3l3*	CAMP Responsive Element Binding Protein 3 like 3 (CREB)	0.0003	1.6623
*Creb3*	CAMP Responsive Element Binding Protein 3 (CREB)	0.0103	0.3544
*Grin1*	Glutamate Iontropic Receptor NMDA Type Subunit 1	0.0000	1.5361
*Kcna6*	Potassium Voltage-gated Channel Subfamily A Member 6	0.0000	0.9101

**Table 2:** Common housekeeping genes are altered in expression in the colon after SCI compared to naive. FDR and logFC were obtained from RNA sequencing.

**Gene**	**Encoded protein**	**Timepoint**	**FDR**	**logFC**
*GAPDH*	Glyceraldehyde-3-Phosphate Dehydrogenase	Day 1	0.1752	−0.2303
		Day 7	0.0000	0.5041
*Actb*	Beta-actin	Day 1	0.0012	−0.4486
		Day 7	0.0001	0.4664
*Tubb5*	Beta-tubulin Class I	Day 1	0.0000	−0.9121
		Day 7	0.0020	0.4596
*Tubb2A*	Beta-tubulin Class II	Day 1	0.0019	−0.5415
		Day 7	0.0000	0.6861
*Tubb3*	Beta-tubulin Class III	Day 1	0.0521	0.9271
		Day 7	0.0000	1.0630
*Tubb4b*	Beta-tubulin Class IV	Day 1	0.0000	−0.9871
		Day 7	0.0003	0.5632

**Fig. 4:**
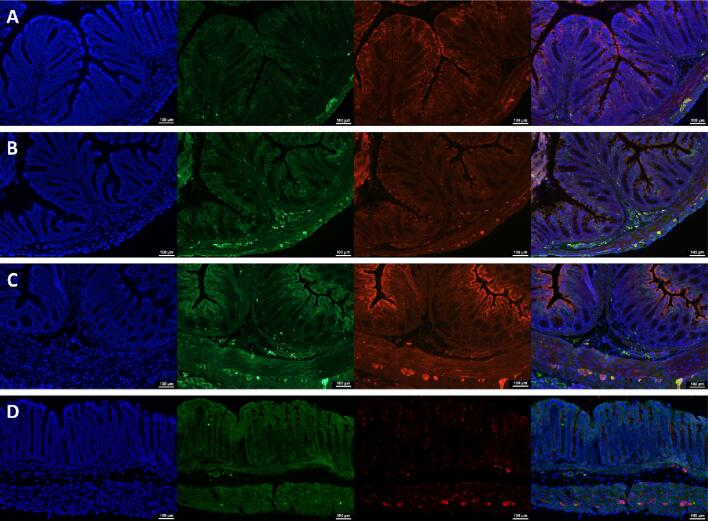
Representative images of immunohistochemistry stain of distal colon cross-sections from naïve **(A)**, D1 **(B)**, D7 **(C),** and D28 (**D**) mice. Sections were stained with DAPI nuclear stain (blue), CGRP (green), PGP9.5 for neurons (red), and merge of all three to show co-localization of CGRP and ENS cell bodies (yellow), n = 5. (For interpretation of the references to color in this figure legend, the reader is referred to the web version of this article.)

In line with our recent report of increased CGRP protein in the periphery (hind paw skin) after SCI (Eller et al., 2022), we leveraged the RNAseq data to specifically assess expression of the two genes known to encode the CGRP protein, *Calca* and *Calcb*. RNAseq showed significantly higher *Calca* expression at D1 (FDR = 0.0280, logFC = 4.7215) and *Calcb* at D7 (FDR = 0.0004, logFC = 1.3202) after SCI. Protein expression of CGRP assessed via immunohistochemistry staining of colon cross-sections confirmed CGRP expression in the colon which reflects the results of the mRNA transcripts (Fig. 4). However, it appears that CGRP protein expression returns to naïve levels at D28 after SCI. While the pathway downstream of CGRP is not significantly altered in the colon 24 h after injury (data not shown), multiple components of the CGRP pathway are significantly increased in the colon seven days after SCI as suggested by the RNAseq (Table 1). This suggests a direct effect of CGRP action in the colon and induction of colonic gene expression changes that may contribute to the development of NB. Of note, in our RNAseq data, we identified significant shifts in expression for housekeeping genes typically used as controls in qPCR and western blotting (e.g. *GAPDH, Actb,* and several genes that encode for β-tubulin; stats reported in Table 2), indicating that these controls may be inappropriate for the calculation of relative mRNA expression values in those assays in SCI animals.

### 3.3 SCI disrupts colon structure and function, allowing bacterial translocation into the gut wall

**Fig. 5:**
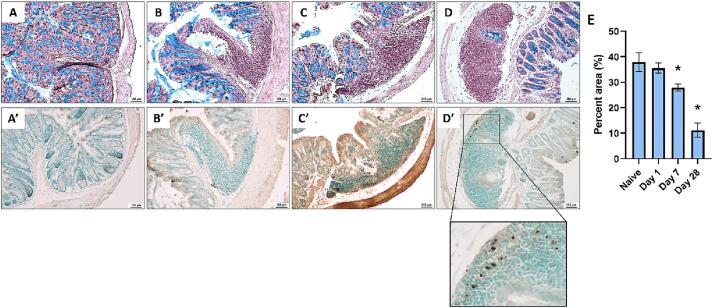
Histology of colon cross-sections suggests expanded lymph nodes, DNA damage, and microbiome translocation into the colon wall. **(A-D)** An Alcian Blue stain showing reduced abundance of goblet cells (mucus producing cell) in the colon, as well as tertiary lymphoid structures as stained by Nuclear Fast Red. Goblet cells are stained with Alcian blue, and sections were counter-stained with Nuclear Fast Red. Representative images of naïve (**A**), D1 (**B**), D7 (**C**), and D28 (**D**). n = 5 per condition. **(E)** Alcian blue was quantified as percent of image area to convey a representation of goblet cell abundance. There was a significant reduction in percent area of Alcian staining for D7 (*p =* 0.021) and D28 (*p <* 0.000) with no significant difference for D1 (*p =* 0.561). The bar graph is depicted as mean ± (SEM). n = 5 per condition. One way ANOVA, LSD post-hoc, *p < 0.05. **(A’-F’)** Terminal deoxynucleotidyl transferase dUTP nick end labeling (TUNEL) stain using HRP-DAB detection and Methyl Green counterstain. DNA damage appears to be localized to the tertiary lymphoid structures as highlighted by the inset in panel D’. Representative images of naïve (**A’**), D1 (**B’**), D7 (**C’**), and D28 (**D’**). N = 5 per condition. (For interpretation of the references to color in this figure legend, the reader is referred to the web version of this article.)

Alcian Blue staining of the colonic goblet cells (which produce mucus) with Nuclear RedFast counterstaining revealed a decrease in abundance of Goblet cells in the colon (Fig. 5A-E) following SCI. This was confirmed by a significant main effect of condition (F(3,16) = 19.064, *p =* 0.000). Post hoc analysis confirmed a significant difference between naïve and D7 (*p =* 0.021) as well as D28 (*p <* 0.000) with no significant difference between naïve and D1 (*p =* 0.561). Moreover, this stain showed a visible expansion of lymph nodes at all timepoints after SCI, which represent tertiary lymphoid structures (TLSs) (Fig. 5A-D). TLSs are typically seen in chronic inflammatory conditions (Hubscher et al., 2018, Schumacher and Thommen, 2022) in agreement with our hypothesis that SCI induces neurogenic inflammation in peripheral tissues, which can be mediated by CGRP.

Terminal deoxynucleotidyl transferase dUTP nick end labeling (TUNEL) staining was used to visualize DNA damage in the colon (Fig. 5A’-D’) and localized the damage to the area of the TLSs and the surrounding epithelial cells. In contrast, DNA damage was visually limited in noninflamed tissues of the colon wall, suggesting that expansion of immune cells and/or an increased inflammatory response by these cells may play a primary role in initiating DNA damage.

**Fig. 6:**
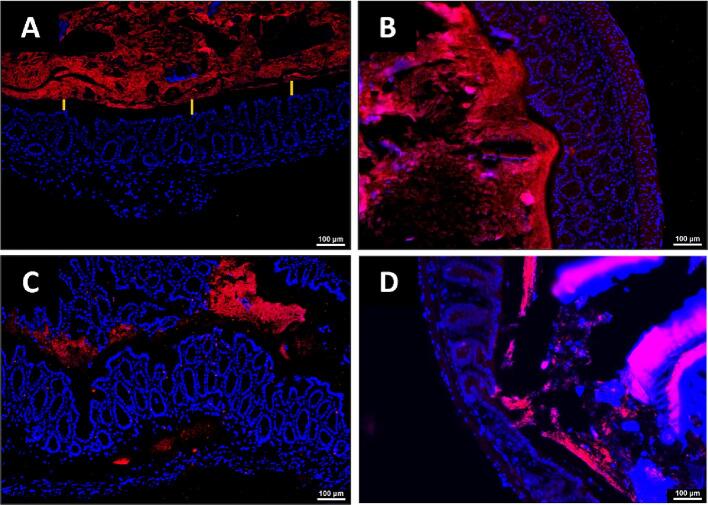
Fluorescent *in situ* Hybridization (FISH) stain showing thinning of sterile mucus later and translocation of microbiota into the colon wall. FISH assay conducted using a DNA oligo probe (EUB338) designed to be complimentary with a generalized bacteria sequence and conjugated with a TEX615-Red fluorophore. Sections are counter-stained with DAPI. The yellow lines represent the sterile mucus layer void of bacteria. Representative images of naïve (**A**), D1 (**B**), D7 (**C**), and D28 (**D**). n = 5 per condition. (For interpretation of the references to color in this figure legend, the reader is referred to the web version of this article.)

Goblet cells are known to produce the protective, barrier mucus that lubricates the gut lumen and prevents lumen contents (e.g. bacteria, fecal material) from coming into direct contact with the colon wall (Buettner and Lochner, 2016). The decreased abundance of goblet cells described above pointed to the potential for decreased integrity of the mucus layer. To assess the functional consequences of reduced goblet cell numbers, *FISH* staining using an EUB338 probe was used to visually localize the microbes within the gut. The colons harvested from naïve mice displayed a thick and uniform barrier devoid of bacteria as expected (Fig. 6A). In contrast, by D1 post-SCI, involutions of the microbes into the inner, sterile mucus layer are visible (Fig. 6B), and by D7, bacteria could be seen translocating into the gut wall as far as the muscle layer (Fig. 6C). By D28 SCI, mucosal integrity resembling the D1 condition with areas of microbial invasion close to the colon wall (Fig. 6D).

Returning to the RNAseq data, we examined transcript abundance for genes known to play a role in the physical component of the intestinal barrier, i.e. genes known to encode for components of mucus and tight junctions (Table 3). *Muc2* encodes for a glycoprotein that makes up the structural component of colonic mucus (Buettner and Lochner, 2016). Transcript expression was not significantly altered at either D1 (FDR = 0.9776, logFC = -0.0979) or D7 (FDR = 0.6769, logFC = -0.2940) compared to naïve. Regarding tight junctions, *Cldn1* was the only altered gene at D1 and was significantly upregulated (FDR = 0.0027, logFC = 1.3832). However, there were two significantly upregulated genes at D7 (*Jam2*, FDR = 0.0470, logFC = 0.4395; *Jam3*, FDR = 0.0000, logFC = 0.8028) with one single gene (*Tjp1*) that was downregulated post-injury (FDR = 0.0117, logFC = -0.7025).

**Table 3:** Tight junction genes are either not altered in expression or significantly increased in the colon after SCI. FDR and logFC were obtained from RNA sequencing.

**Gene**	**Encoded protein**	**Timepoint**	**FDR**	**logFC**
*Ocln*	Occludin	Day 1	0.9272	−0.0556
		Day 7	0.9679	0.0068
*Jam2*	Junction Adhesion 2	Day 1	0.3979	0.2734
		Day 7	0.0470	0.4395
*Jam3*	Junction Adhesion 3	Day 1	0.1112	0.3445
		Day 7	0.0000	0.8028
*Cldn1*	Claudin 1	Day 1	0.0027	1.3832
		Day 7	0.2649	0.5437
*Tjp1*	Tight Junction Protein 1/Zona Occludens-1	Day 1	0.5414	0.2768
		Day 7	0.0117	−0.7025
*Tjp2*	Tight Junction Protein 2	Day 1	0.9317	0.0443
		Day 7	0.4325	0.1478
*Tjp3*	Tight Junction Protein 3	Day 1	0.8256	0.0929
		Day 7	0.5851	0.1308

### 3.4 SCI evokes gut dysbiosis and microbe-induced host gene expression changes

**Fig. 7:**
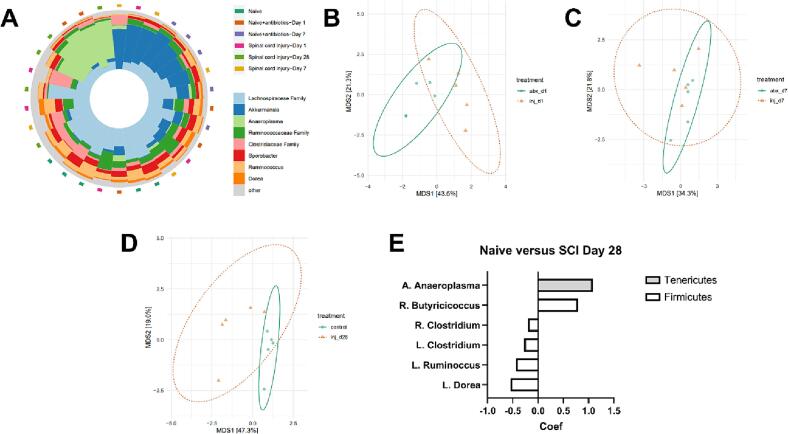
16S rRNA sequencing reveals significant alterations in the gut microbiome composition. **(A)** Taxonomic Iris Plot of the relative abundance of microbial families per mouse per condition. **(B-D)** Ordination diagrams of Principal Components Analysis (MDS) show significant differences in group distance as calculated with Aitchison distance. Acute SCI timepoints (D1 SCI in panel B and D7 SCI in panel C) are compared to antibiotic controls while D28 SCI is compared to naïve baseline. D1 SCI (*p =* 0.02) and D28 SCI (*p =* 0.009) are significantly different than their respective controls whereas D7 SCI is not (*p =* 0.169). Significance was calculated using a permutational multivariate ANOVA, n = 5 per condition, *p <* 0.05. **(E)** Histogram plot of microbial families with significant coefficient values at D28 SCI compared to naïve. There is overall a significant decrease in Firmicutes and increase in Tenericutes. Significant taxa are selected based on *p <* 0.1, n = 5 per condition.

The observed bacterial translocation after SCI suggests a role for these gut microbes in the pathogenesis in NB, however this analysis does not provide any clarity on the composition of the microbial communities translocating out of the gut lumen. 16S rRNA sequencing of the fecal microbiome was used to assess shifts in bacterial colonization of the gut microbiome after SCI (Fig. 7). In order to prevent post-operative infections, the mice were treated with a low dosage of Gentamicin Sulfate for five days after SCI. To control for the effect of antibiotic treatment on the acute timepoints, we compared D1 SCI and D7 SCI to antibiotic-treated, unijured controls at each respective timepoint. The pairwise Aitchison distance, as calculated from Principal Component Analysis (PCoA), showed significant differences in global composition for D1 post-SCI (Fig. 7B, *p =* 0.02) and D28 SCI (Fig. 7D, *p =* 0.009). Analysis with *MaAsLin2* allowed us to assess specific taxa at the phylum, family, and genus levels. D1 post-SCI, a single operational taxonomic unit (OTU), *A. Anaeroplasma* of the *Tenericutes* phylum, was significantly decreased (coef = -1.905, *p =* 0.0005). By D28, a number of different OTUs differed significantly from the control (Fig. 7E). Most notably in the D28 SCI group, a significant increase in a potentially pathogenic, gram-negative microbe in the *Tenericutes* phylum (*A. Anaeroplasma*) was noted as well as a significant decrease in several commensal microbes belonging to the *Firmicutes* phylum.

**Fig. 8:**
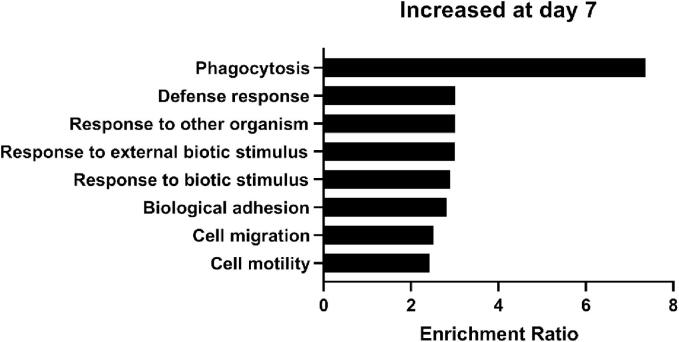
Gene Ontology terms for upregulated mRNA transcripts in the below-level DRG D7 after SCI. These GO terms suggest bacteria-induced host gene expression changes in below-level spinal neurons.

Identification of gut microbiome composition changes and microbial translocation out of the lumen point to the potential for host-microbiome interactions in the early pathogenesis of NB. It was unclear from these other analyses if these bacteria are directly interacting with host cells. We, therefore, sorted through RNAseq of the colon in combination with RNAseq of DRG from below the level of the SCI, which contain the primary sensory neurons that innervate the hindgut. We searched for transcriptional indicators of microbial interactions by assessing the expression of the primary neuronal receptors for gram-negative and gram-positive bacteria, Toll-like receptor 4 and Toll-like receptor 2, respectively. In the colon, Toll-Like Receptor 4 (*Tlr4*) transcript abundance is not significantly different at D1 (FDR = 0.8510, logFC = -0.1070) or D7 (FDR = 0.5109, logFC = -0.1820) post-SCI. In contrast, while Toll-Like Receptor 2 (*Tlr2*) transcript abundance was not significantly altered at D1 after SCI (FDR = 0.8658, logFC = 0.1042), it was significantly increased by D7 (FDR = 0.0359, logFC = 0.5257). TLR activation by lipopolysaccharide (LPS; cell wall component of gram-negative bacteria) induces pro-inflammatory cytokine expression and release *in vivo* and *in vitro* (Johansson et al., 2008). A similar approach was applied to the investigation of transcript abundance in below-level DRG after injury, with the goal of determining whether gut microbes were communicating with primary sensory neurons involved in the transmission of sensory information from the bowel and could therefore play a role in sensory dysfunction. GO terms associated with the response to bacterial stimulation reflected enrichment of specific genes within the gene set (Fig. 8). *Tlr2* was significantly higher by D7 after SCI (FDR = 0.0065, logFC = 1.2132) as were other genes associated with bacterial stimulation (i.e. Lipopolysaccharide Binding Protein *(Lpb),* Peptidoglycan Recognition Protein 1 *(Pglyrp1),* Cathelicidin Antimicrobial Peptide *(Camp),* etc).

### 3.5 SCI alters Ca^2+^ transients of below-level colon specific DRG and ND extrinsic primary afferents to High K^+^ and capsaicin

Quantifying the visceromotor response (VMR) to colorectal distension is a validated technique to assess visceral hypersensitivity (VH) (Kigerl et al., 2007). We expect that SCI induces VH based on clinical reports of chronic abdominal pain after SCI, however, SCI abolishes the ability for rodents to generate the spinobulbospinal reflex required for to generate a VMR (Kigerl et al., 2007). Given the technical challenge of behaviorally assessing VH in our model with the knowledge that an inability to generate a behavioral response does not necessarily indicate an absence of pain transmission from the colon, we leveraged the response profile of colon-specific primary afferent populations as a proxy measure of increased responsiveness to noxious stimuli after SCI. Both gut dysbiosis and the inflammatory microenvironment of the colon have been shown to alter primary sensory neuronal sensitivity/activity. Prior reports by several groups reveal differences in the electrophysiological properties and calcium (Ca^2+^) transients of extrinsic spinal (DRG) (Kamp et al., 2003, Ochoa-Cortes et al., 2010) and vagal (ND) electrical excitability (Hou and Wang, 2001) in response to stimulation with LPS from gram-negative bacteria. However, these experimental results were not assessed within the context of neurogenic bowel. To accomplish this, we assessed Ca^2+^ transients in cultured DRG and ND neurons in response to stimulation with various agonists, including fecal supernatants. We also examined calcium transients in intrinsic (enteric nervous system, ENS) neurons isolated from the myenteric plexus of the colon. Unlike the DRG and ND which contain only sensory neuron cell bodies, the ENS preparation includes intrinsic primary afferents, interneurons, and motor neurons, but examining the ENS can shed light on alterations in intrinsic neurons with the potential to affect abdominal pain sensation and/or dysmotility.

**Fig. 9:**
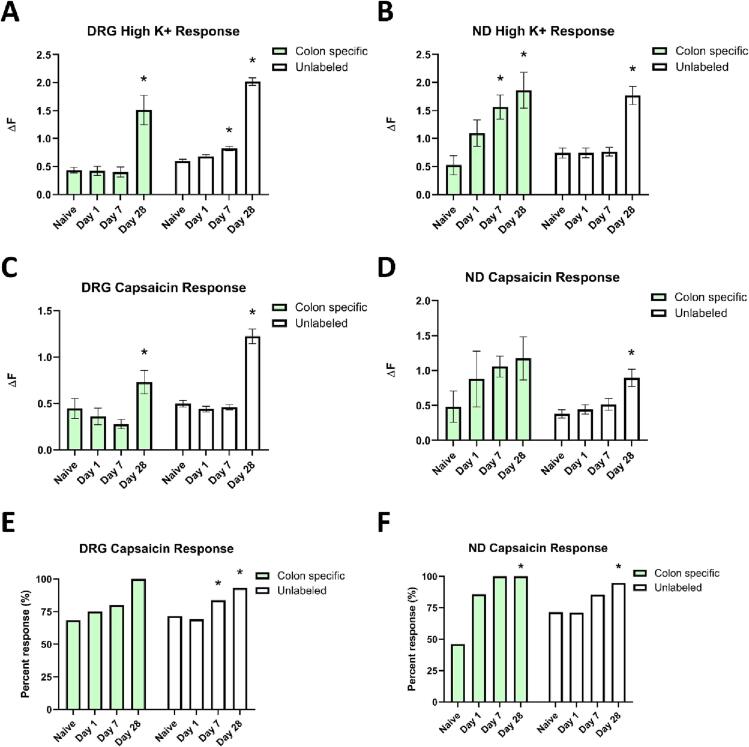
Calcium imaging of retrograde-labeled, colon specific or unlabeled dorsal root ganglion (DRG) and nodose ganglion (ND) primary afferents reveals increased responsiveness to High K^+^ and capsaicin after SCI. **(A,B)** High K^+^ was used to treat DRG and ND cells during calcium imaging. There was a significant increase in the ΔF for unlabeled DRG at D7 (*p =* 0.005) and D28 compared to naïve (*p =* 0.001) as well as a significant increase for D28 compared to naïve for colon specific cells (*p =* 0.001). N = 3 mice per condition, two-way ANOVA, one-way ANOVA within each cell population, LSD post-hoc, *p < 0.05. There was a significant increase in the ΔF for unlabeled ND at D28 compared to naïve (*p =* 0.001) as well as a significant increase for D7 (*p =* 0.025) and D28 (*p =* 0.001) compared to naïve for colon specific cells. N = 3 mice per condition, two-way ANOVA, one-way ANOVA within each cell population, LSD post-hoc, *p < 0.05. **(C,D)** DRG and ND were treated with 1 μM capsaicin to activate TRPV1 ion channels and measured calcium influx. There was a significant increase in the ΔF for unlabeled DRG at D28 compared to naïve (*p =* 0.001) as well as for colon specific cells (*p =* 0.049). N = 3 mice per condition, two-way ANOVA, one-way ANOVA within each cell population, LSD post-hoc, *p < 0.05. For capsaicin treatment, there was a significant increase in the ΔF for unlabeled ND at D28 compared to naïve (*p =* 0.001) but not for colon specific cells. N = 3 mice per condition, two-way ANOVA, one-way ANOVA within each cell population, LSD post-hoc, *p < 0.05. **(E, F)** There was a significant increase in the proportion of cells that responded to capsaicin for unlabeled DRG cells (*p =* 0.001) but was not significantly different for colon specific cells (*p =* 0.065). Individual χ^2^-square tests confirmed a significant increase in proportion for D7 (*p* = 0.001) and D28 (*p* < 0.001) compared to naïve. N = 3 mice per condition, χ^2^-Square test, *p < 0.05. In addition, there was a significant increase in the proportion of cells that responded to capsaicin for unlabeled ND cells (*p =* 0.001) and for colon specific cells (*p =* 0.010). Individual χ^2^-square tests confirmed a significant increase in proportion for the D28 for both colon specific (*p* = 0.005) and unlabeled (*p* < 0.001) cells compared to their respective naïve controls. N = 3 mice per condition, χ^2^-Square test, *p < 0.05.

Ca^2+^ imaging was employed to determine Ca^2+^ transient changes in DRG and ND extrinsic primary afferents that innervate the distal colon (retrogradely labeled; CBT + ), as well as unlabeled neurons. Neurons were exposed to 30 mM (High) K^+^ to assess neuronal health and measure the peak Ca^2+^ influx (ΔF) in response to depolarization (Fig. 9A,B). A 2 x 4 ANOVA was conducted to determine the effect of cell population (CTB + vs unlabeled) and condition (naïve, D1, D7, and D28) on DRG ΔF (Fig. 9A). It confirmed a significant main effect of cell population (F (1,1218) = 12.793, *p <* 0.001) and a significant main effect of condition (F (3,1218) = 39.388, *p <* 0.001), however there was no significant interaction (F (3,1218) = 0.766, *p =* 0.513). We next conducted follow up one-way ANOVAs within each cell population to clearly isolate which conditions were driving the significant overall effects. For the colon specific CTB + DRG neurons, there was a significant main effect of condition (F (3,91) = 17.395, *p =* 0.001). Post hoc analysis confirmed this main effect was the result of a significant increase in peak Ca^2+^ influx at D28 (*p =* 0.001) compared to all other conditions. In unlabeled DRG neurons, there was a significant main effect of condition (F (3,1127) = 194.432, *p =* 0.001). Post hoc analysis confirmed a significant increase in peak Ca^2+^ influx for D7 (*p =* 0.005) as well as D28 DRG neurons (*p =* 0.001), compared to naive.

For the ND cells (Fig. 9B), a 2 x 4 ANOVA confirmed a significant main effect of condition (F (3,249) = 10.832, *p <* 0.001), however there was not a significant main effect of cell population (F (1,249) = 1.928, *p =* 0.166) or significant interaction (F (3,249) = 1.253, *p =* 0.291). A significant main effect of condition was observed for both CTB+ (F (3,30) = 6.070, *p =* 0.002) and unlabeled ND neurons (F (3,219) = 19.178, *p =* 0.001). Post hoc analyses confirmed this was the result of a significant increase in peak Ca^2+^ influx in D7 (*p =* 0.025) and D28 (*p =* 0.001) in CTB + and D28 (*p =* 0.001) in non-labeled ND neurons, compared to their respective naïve populations.

We next assessed peak Ca^2+^ influx in response to 1 µM capsaicin as an indication of putative nociceptor function (Fig. 9C-F). For the ΔF for the DRG cells (Fig. 9C), 2 x 4 ANOVA confirmed a significant main effect of condition (F (3,975) = 6.682, *p <* 0.001), however there was not a significant main effect of cell population (F (1,975) = 3.234, *p =* 0.072) or significant interaction (F (3,975) = 0.916, *p =* 0.432). We conducted follow up one-way ANOVAs within each cell population. For the colon specific DRG cells, there was a significant main effect of condition (F (3,70) = 2.940, *p =* 0.039). Post hoc analysis confirmed a significant increase only in D28 (*p =* 0.049). For the ΔF for the unlabeled DRG neurons, there was a significant main effect of condition (F (3,905) = 48.199, *p =* 0.001). Post hoc analysis confirmed a significant increase in the responsiveness in D28 (*p =* 0.001) compared to naive.

For the ND cells (Fig. 9D), a 2 x 4 ANOVA confirmed a significant main effect of condition (F (3,203) = 2.922, *p =* 0.035) and a significant main effect of cell population (F (1,203) = 4.140, *p =* 0.043), however there was no significant interaction (F (3,203) = 0.292, *p =* 0.831). We conducted follow up one-way ANOVAs within each cell population. For the colon specific ND cells, there was not a significant main effect of condition (F (3,22) = 0.835, *p =* 0.489). For the unlabeled ND cells, there was a significant main effect of condition (F (3,181) = 5.350, *p =* 0.001). Post hoc analysis confirmed a significant increase in D28 responsiveness (*p =* 0.001).

The proportion of DRG (Fig. 9E) and ND neurons (Fig. 9F) that responded to 1 µM capsaicin was calculated for each timepoint. A χ^2^ test determined that there was no significant change in proportion for CTB + DRG neurons that responded to capsaicin (X^2^ = 7.244, *p =* 0.065). However, the proportion of unlabeled DRG neurons that responded was significantly higher in SCI groups than naive (χ^2^ = 71.227, *p =* 0.001), driven by a higher proportion of capsaicin responsive neurons in D7 (χ^2^ = 10.474, *p =* 0.001) and D28 (χ^2^ = 47.058, *p <* 0.001) compared to the naïve condition. The proportion of capsaicin responsive CTB + ND neurons was significantly higher in SCI groups than naive (χ^2^ = 11.281, *p =* 0.010), driven by a significant increase in observed relative to expected counts in the D28 group compared to naïve (χ^2^ = 7.740, *p =* 0.005), however comparisons of D1 (χ^2^ = 2.967, *p =* 0.085) and D7 (χ^2^ = 3.662, *p =* 0.056) to the naïve condition only approached significance. The proportion of unlabeled ND neurons that responded to capsaicin was significantly higher in SCI compared to naïve (χ^2^ = 16.290, *p =* 0.001), driven by higher proportions of observed vs expected counts in the D28 group compared to naïve (χ^2^ = 12.897, *p <* 0.001).

### 3.6 SCI alters the Ca^2+^ transients of enteric neurons to High K^+^ but not capsaicin

**Fig. 10:**
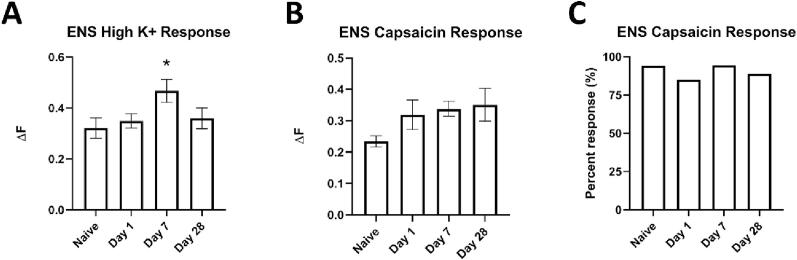
Calcium imaging of enteric nervous system neurons reveals increased responsiveness to High K^+^ but not capsaicin. **(A)** ENS cells were assessed by treatment of High K^+^. There was a significant increase in the ΔF for D7 SCI compared to naïve (*p =* 0.017). N = 3 mice per condition, one-way ANOVA, LSD post-hoc, *p < 0.05. **(B,C)** For capsaicin treatment, there was no significant increase in the ΔF for any timepoint compared to naïve. N = 3 mice per condition, one-way ANOVA, LSD post-hoc, *p < 0.05. In addition, there was no significant difference in the proportion of cells that responded to capsaicin. N = 3 mice per condition, χ^2^-Square test, *p < 0.05.

Ca^2+^ imaging was also used to determine functional changes in intrinsic neurons within the enteric nervous system (ENS) due to SCI (Fig. 10). A significant main effect of condition (F (3,280) = 2.741, *p =* 0.044) was observed for peak Ca^2+^ influx in response to High K^+^ (Fig. 10A). This effect was driven by a significant difference in peak Ca^2+^ influx between naïve and D7 (*p =* 0.017). No significant main effect of condition was observed for peak Ca^2+^ influx in response to capsaicin (F (3,254) = 0.140, *p =* 0.166, Fig. 10B). Similarly, a χ^2^ test determined that the proportion of capsaicin-responsive cells was not significantly altered after SCI (χ^2^ = 5.649, *p =* 0.138, Fig. 10C).

### 3.7 Gut dysbiosis induced by SCI alters the Ca^2+^ transients of both extrinsic and intrinsic primary afferents to autologous fecal supernatants

**Fig. 11:**
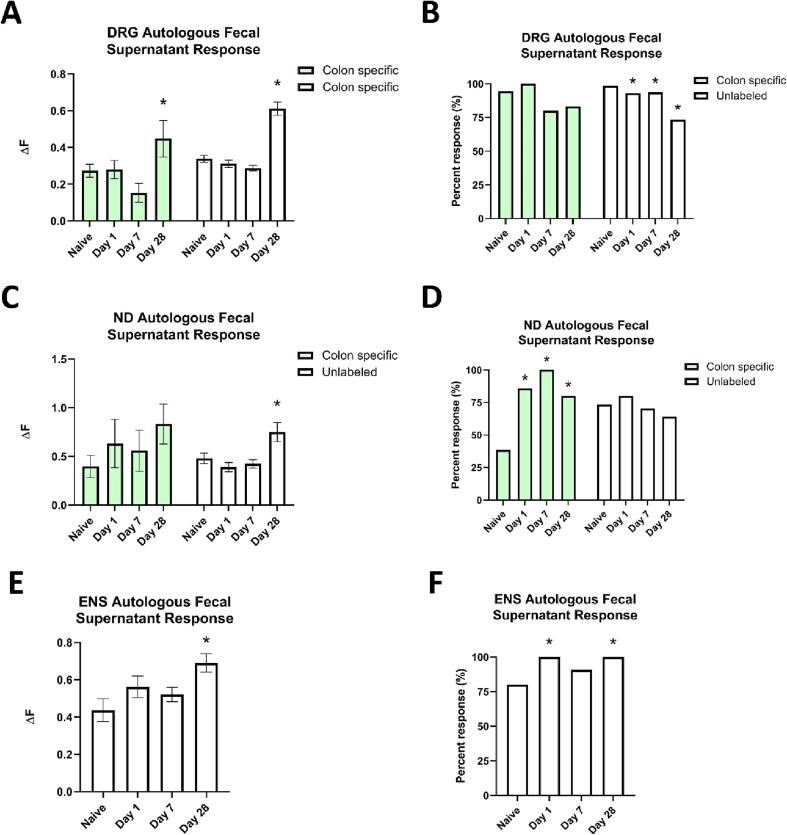
Calcium imaging of retrograde-labeled, colon specific or unlabeled dorsal root ganglion (DRG; Panels A and B) and nodose ganglion (ND; Panels C and D) primary afferents, as well as enteric nervous system neurons (ENS; Panels E and F). Cells were treated with autologous gut lumen contents to reveal significant alterations in neuronal responsiveness to microbiome shifts induced by SCI. **(A, B)** There was a significant increase in the ΔF for unlabeled DRG at D28 compared to naïve (*p =* 0.001) as well as for colon specific cells (*p =* 0.029). N = 3 mice per condition, two-way ANOVA, one-way ANOVA within each cell population, LSD post-hoc, *p < 0.05. In addition, there was a significant decrease in the proportion of cells that responded to autologous gut lumen contents for unlabeled DRG cells (*p =* 0.001) but was not significantly different for colon specific cells (*p =* 0.084). N = 3 mice per condition. For the unlabeled cells, individual χ^2^-square tests confirmed a significant decrease in proportion for D1 (*p* = 0.005), D7 (*p* = 0.010), and D28 (*p* < 0.001) compared to naive. χ^2^-Square test, *p < 0.05. **(C, D)** There was a significant increase in the ΔF for unlabeled ND at D28 compared to naïve (*p =* 0.007) but not for colon specific cells N = 3 mice per condition, two-way ANOVA, one-way ANOVA within each cell population, LSD post-hoc, *p < 0.05. In addition, there was not a significant increase in the proportion of cells that responded to autologous gut lumen contents for unlabeled ND cells but there was a significant increase for colon specific cells (*p =* 0.033). Individual χ^2^-square tests confirmed a significant decrease in proportion for D1 (*p* = 0.043), D7 (*p* = 0.031), and D28 (*p* = 0.046) compared to naive. N = 3 mice per condition, χ^2^-Square test, *p < 0.05. **(E, F)** There was a significant increase in the ΔF for ENS at D28 compared to naïve (*p =* 0.004). N = 3 mice per condition, one-way ANOVA, LSD post-hoc, *p < 0.05. In addition, there was a significant increase in the proportion of cells that responded to autologous gut lumen contents for ENS cells (*p =* 0.001). Individual χ^2^-square tests confirmed a significant decrease in proportion for D1 (*p* < 0.001) and D28 (<0.001) compared to naive. N = 3 mice per condition, χ^2^-Square test, *p < 0.05.

The observed changes in colonic microbial colonization and the corresponding effects on neuronal gene expression suggest potential direct interactions between extrinsic primary afferents and the contents of the gut lumen but do not assess alterations in neuronal responses *per se*. Prior work indicates that microbes and their related factors (e.g. microbial metabolites) are able to induce neuronal response changes and are shown to be associated with pain states (Ochoa-Cortes et al., 2010, Pradhananga et al., 2020). We therefore exposed CTB + DRG and ND cells, as well as intrinsic enteric neurons, to fecal supernatants from the same mouse from which the cells were harvested (i.e. autologous gut lumen contents). We quantified both peak influx of Ca^2+^ (ΔF) and proportion of cells that responded to the autologous gut lumen contents (Fig. 11).

A 2 x 4 ANOVA of the ΔF for DRG cells (Fig. 11A) confirmed a significant main effect of condition (F (3,1119) = 7.325, *p <* 0.001) and a significant main effect of cell population (F (1,1119) = 5.032, *p =* 0.025), however there was no significant interaction (F (3,1119) = 0.468, *p =* 0.705). We next conducted follow up one-way ANOVAs within each cell population. For the colon specific DRG cells, there was a significant main effect of condition (F (3,86) = 3.372, *p =* 0.022). Post hoc analysis confirmed a significant difference for D28 (*p =* 0.029) compared to naive. In unlabeled DRG cells, there was a significant main effect of condition (F (3,1033) = 40.045, *p =* 0.001) on ΔF. Post hoc analysis confirmed a significant difference increase in responsiveness for D28 (*p =* 0.001) compared to naive. A χ^2^-Square test determined that the proportion of cells that responded to autologous gut lumen contents was not significantly different for colon specific DRG cells (χ^2^ = 6.651, *p =* 0.084) but was significantly altered for unlabeled DRG (χ^2^ = 107.244, *p =* 0.001) driven by reduced response for all post-SCI timepoints compared to naïve (D1, χ^2^ = 7.944, *p =* 0.005; D7, χ^2^ = 6.657, *p =* 0.010; D28, χ^2^ = 55.512, *p <* 0.001; Fig. 11B).

For the ND (Fig. 11C), a 2 x 4 ANOVA confirmed a significant main effect of condition (F (3,173) = 2.823, *p =* 0.040), however there was neither a significant main effect of cell population (F (1,173) = 0.794, *p =* 0.374) nor a significant interaction (F (3,173) = 0.404, *p =* 0.750). We next conducted follow up one-way ANOVAs within each cell population. For the colon specific ND cells, there was not a significant main effect of condition (F (3,19) = 0.205, *p =* 0.521), but there was a significant main effect of condition for the unlabeled ND cells (F (3,154) = 5.811, *p =* 0.001). Post hoc analysis confirmed a significant increase for D28 (*p =* 0.007). The proportion of cells that responded was significantly higher for the colon specific ND cells (χ^2^ = 8.714, *p =* 0.033) driven by a lower proportion of naïve cells compared to all timepoints after SCI (D1, χ^2^ = 4.105, *p =* 0.043; D7, χ^2^ = 4.650, *p =* 0.031; D28, χ^2^ = 3.969, *p =* 0.046; Fig. 11D). However, it was not significantly different for unlabeled ND (χ^2^ = 3.697, *p =* 0.296, Fig. 11D).

For isolated ENS cells (Fig. 11E), there was a significant main effect of condition (F (3,260) = 3.200, *p =* 0.024), and a post hoc analysis confirmed a significant increase for D28 compared to naive (*p =* 0.004). The proportion of ENS cells that responded was significantly higher following SCI (χ^2^ = 23.181, *p =* 0.001) driven by a lower proportion of naïve cells compared to D1 (χ^2^ = 15.892, *p <* 0.001) and D28 (χ^2^ = 11.740, *p <* 0.001; Fig. 11F) with D7 trending towards significance (χ^2^ = 3.566, *p =* 0.059).

### 3.8 Fecal supernatant from D28 SCI rodents is sufficient to induce Ca^2+^ transients of both extrinsic and intrinsic naïve primary afferents

**Fig. 12:**
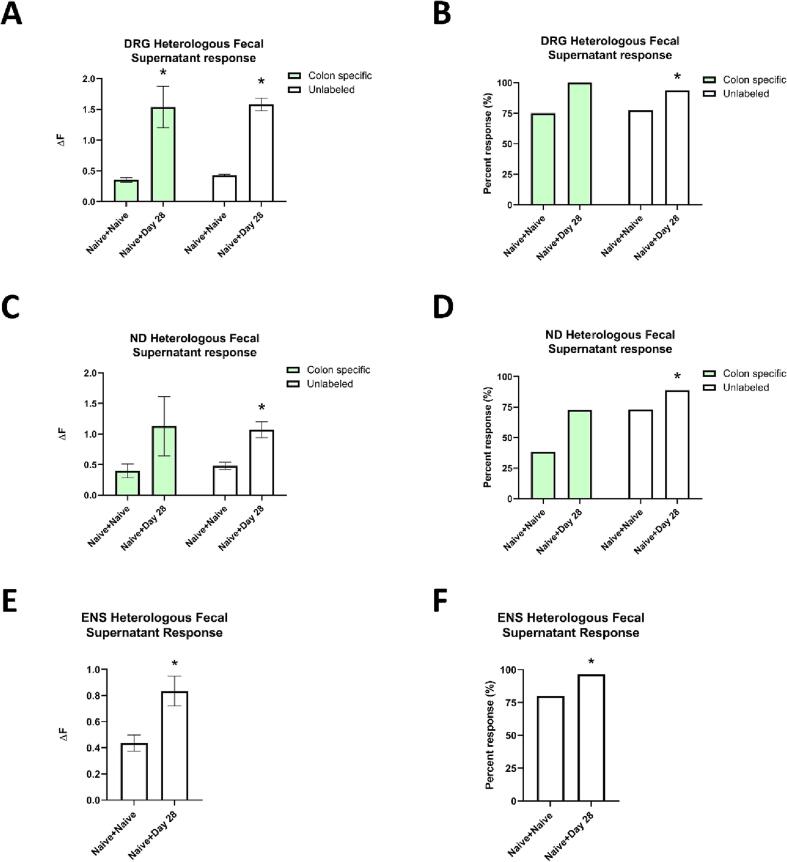
Calcium imaging of retrograde-labeled, colon specific or unlabeled dorsal root ganglion (DRG; Panels A and B) primary afferents, nodose ganglion (ND; Panels C and D) primary afferents, and intrinsic enteric neurons (ENS; Panels E and F). Cells were treated with gut lumen contents from naïve mice or D28 SCI mice to reveal significant alterations in neuronal responsiveness to microbiome shifts independently of SCI. **(A, B)** There was a significant increase in the ΔF for naive unlabeled DRG cells treated with D28 SCI fecal supernatant (*p =* 0.001) as well as for colon specific DRG cells (*p =* 0.001). N = 3 mice per condition, two-way ANOVA, independent samples *t*-tests within cell populations, *p < 0.05. In addition, there was a significant increase in the proportion of cells that responded for unlabeled DRG cells (*p =* 0.001) but no change in colon specific cells. N = 3 mice per condition, Pearson χ^2^-Square test, *p < 0.05. **(C, D)** There was a significant increase in the ΔF for naive unlabeled ND cells treated with D28 SCI fecal supernatant (*p =* 0.007) but not for colon specific ND cells. N = 3 mice per condition, two-way ANOVA, independent samples *t*-tests within cell populations, *p < 0.05. In addition, there was a significant increase in the proportion of cells that responded for unlabeled ND cells (*p =* 0.011) but no change in colon specific cells. N = 3 mice per condition, Pearson χ^2^-Square test, *p < 0.05. **(E, F)** There was a significant increase in the ΔF for naive ENS cells treated with D28 SCI fecal supernatant (*p =* 0.048). N = 3 mice per condition, independent samples *t*-test, *p < 0.05. In addition, there was a significant increase in the proportion of cells that responded to D28 SCI stool (*p =* 0.001). N = 3 mice per condition, Pearson χ^2^-Square test, *p < 0.05.

Due to the observed increase in primary afferent responsiveness to autologous gut lumen contents, we attempted to determine whether this phenotype resulted directly from spinal injury or if it was mediated by the dynamic shift in microbiome composition. To evaluate this, we performed Ca^2+^ imaging on colon specific (CTB + ), DRG and ND primary afferents from naïve mice, as well as intrinsic enteric neurons. All neuronal cultures were then treated with fecal supernatants from D28 SCI mice (i.e., heterologous gut lumen contents) compared to the control condition of treatment with autologous gut lumen content (i.e. from naïve mice). We selected D28 SCI fecal supernatants over other timepoints because of our findings from the microbiome sequencing experiment showing D28-SCI mice exhibit a persistent chronic microbiome shift not seen for the acute timepoints and due to the clinical relevance given that NB is a chronic condition associated with pain that develops over a more protracted timeline. Similar to the first fecal supernatant imaging experiment, we quantified both ΔF, followed by an independent samples *t*-test within each cell population, and the proportion of cells that responded to the autologous gut lumen contents was also assessed (Fig. 12).

Analysis of ΔF for DRG cells (Fig. 12A) with a 2 x 4 ANOVA confirmed a significant main effect of condition (F (3,371) = 43.128, *p <* 0.001), however there was neither a significant main effect of cell population (F (1,371) = 0.108, *p =* 0.743) nor a significant interaction (F (3,371) = 0.007, *p =* 0.933). For the colon specific cells, there was a significant increase in the naïve cells treated with D28 SCI fecal supernatants (*t* = -5.219, *p =* 0.001) compared to those treated with naïve fecal supernatants, as well as for the unlabeled DRG cells (*t* = -10.459, *p =* 0.001). A χ^2^-Square test determined that the proportion of cells that responded was not significantly different for colon specific DRG cells (χ^2^ = 3.692, *p =* 0.055) but was significantly higher for unlabeled DRG (χ^2^ = 20.999, *p =* 0.001, Fig. 12B).

Analysis of ΔF for the ND cells (Fig. 12C), a 2 x 4 ANOVA showed a significant main effect of condition (F (3,145) = 3.892, *p =* 0.050), however there was not a significant main effect of cell population (F (1,145) = 0.001, *p =* 0.973) or a significant interaction (F (3,145) = 0.045, *p =* 0.832). There was not a significant difference in responsiveness for colon specific ND cells (*t* = -1.163, *p =* 0.270), but there was a significant increase in the unlabeled ND cell responsiveness to D28 SCI fecal supernatants (*t* = -2.725, *p =* 0.007). The proportion of cells that responded was not a significantly different proportion for colon specific ND cells (χ^2^ = 2.818, *p =* 0.093) but there was a significantly higher proportion for unlabeled ND (χ^2^ = 6.501, *p =* 0.011, Fig. 12D). For the ENS, there was a significant increase in responsiveness to D28 fecal supernatants (*t* = -1.989*p =* 0.048, Fig. 12E), as well as a significantly higher proportion for those treated with D28 SCI fecal supernatants (χ^2^ = 11.817, *p =* 0.001, Fig. 12F).

## 4 Discussion

NB is a common complication of various disorders of the central nervous system and affects more than 60 % of SCI patients (Faaborg et al., 2013, Finnerup et al., 2008, Krogh et al., 2000), making SCI a promising model for preclinical studies of NB following trauma to the CNS. NB is characterized by severe abdominal pain and bowel dysfunction, which are reported by patients to be a higher priority than the loss of motor function. To date, the majority of clinical studies are observational reports of these symptoms with little insight into the underlying mechanisms. In tandem, most rodent SCI pain research focuses on neuropathic or musculoskeletal pain creating a paucity of established and validated rodent models of NB. This gap in the literature represents a major challenge in generating and testing hypotheses focused on the underlying mechanisms of NB after SCI or in other disorders.

To fill in this knowledge gap, we established the presence of prominent NB-like phenotypes in a translational mouse model of moderate severity T9 contusion SCI. We focused on the early-onset transcriptomic as well as colonic structural and functional changes reflecting potential mechanisms underlying NB initiation and maintenance. Further characterization of gut-specific changes using multi-omics approaches showed increased transcript abundance for *Calca* and *Calcb* which encode for the α and β isoforms of CGRP, respectively. Furthermore, we identified genomic instability represented by DNA damage accumulation, reduced goblet cell abundance, and lymphatic nodule expansion within the colon wall, i.e. tertiary lymphoid structures (TLSs). TLSs are typically associated with chronic inflammatory conditions such as cancer and autoimmunity, (Hubscher et al., 2018, Schumacher and Thommen, 2022) which supports the idea of intestinal inflammation after SCI. Finally, the presence of colonic damage corresponds to late-onset microbiome composition shifts, bacterial translocation into the colon wall, and vagal/enteric nervous system hyperresponsiveness.

### 4.1 Antidromic activity induces neurogenic inflammation through CGRP overexpression

During and after a peripheral injury (e.g. in innervated tissue), electrical signals propagate orthodromically (i.e. from the periphery towards the central nervous system) (Minerbi et al., 2019). However, primary afferents can generate and propagate electric signals bi-directionally (Kress et al., 1999, Peirs and Seal, 2016, Sorkin et al., 2018). When the neuron is injured or stimulated more proximally to the spinal cord, antidromic activity has been shown to stimulate the release of neurotransmitters, hormones, and pro-inflammatory molecules from the terminal endings into the innervated, uninjured tissue (Walters, 2012, Peirs and Seal, 2016, Sluka, 1996). SCI has been shown to induce antidromic activity as well as neurogenic inflammation in peripheral tissues mediated by release of CGRP from peripheral afferents into the skin (Eller et al., 2022). CGRP initiates neurogenic inflammation by activating the inflammasome, causing vasodilation, edema, and immune cell infiltration (Carlton et al., 2009, Fu et al., 2017). We found rapid and persistent expansion of lymphatic nodules in the colon and immune cell infiltration of the colon wall after SCI. Under normal circumstances, pro-inflammatory cells invade a site of injury to eliminate pathogens, damaged cells, and cellular debris to promote wound healing (Kee et al., 2018, Rust and Kaiser, 2017). Following SCI, this response is augmented leading to persistent inflammation in uninjured organs (such as the colon) leading to DNA damage from free radicals, organ dysfunction, and impaired recovery of function (Monteiro et al., 2018).

Following SCI, expression of mRNA transcripts encoding for CGRPα and CGRPβ isoforms in the colon reflected a peak in the α isoform (D1 post-SCI) followed by a peak in the β isoform (D7 post-SCI). There is 94 % homology between the two isoforms even though they are encoded by two separate genes (*Calca* and *Calcb*) (Sun et al., 2016) corresponding to CGRPα primarily restricted to primary afferents and CGRPβ found primarily in intrinsic sensory neurons of the ENS (Fu et al., 2017). Interestingly, *Calca* mRNA can be locally translated outside of the cell body (e.g. in axons/terminals) of primary sensory neurons (Russell et al., 2014, Gale et al., 2022), taken together with our RNAseq data this suggests the increased *Calca* transcript abundance detected in the colon likely reflects *Calca* localized to free nerve endings of colonic DRG neurons. Taken together with prior findings of elevated CGRP in the skin following SCI (Eller et al., 2022), these data point to a critical role for antidromic activity in primary afferents inducing acute expression and release of CGRPα from terminals in close proximity to ENS neurons and leading to subsequent expression of CGRPβ therein. Moreover, both CGRP isoforms induce synthesis and release of nitric oxide (NO), a primary signaling molecule for the inhibitory ENS (Toth et al., 2009) providing a molecular mechanism by which CGRP could inhibit peristalsis, in line with the clinical presentation of NB and the present preclinical data. Further, NMDA receptors have been shown to be involved in visceral nociception and hypersensitivity (Yip et al., 2022), vagal afferent hyperexcitability (McRoberts et al., 2001), and are associated with functional abdominal pain/irritable bowel syndrome (Vance et al., 2015). NMDA receptor expression and activity can be altered by CGRP signaling through RAMP1 (Iyengar et al., 2017). Transcript abundance for both *Ramp1* and *Grin1,* which encodes an NMDA receptor subunit, *were more abundant in the colon after SCI.* Taken together with the known role of NO in ENS inhibition, we hypothesize that CGRP plays a central role in the pathogenesis of both NB-associated abdominal pain and colonic dysmotility.

### 4.2 Dynamic shifts in microbiome composition after SCI

The gut microbiome is a complex environment comprised of diverse bacteria that produce beneficial metabolites (e.g. short chain fatty acids, SCFA) critical to colon structure and function (Willits et al., 2021). Although there is not a strictly defined “eubiotic” or healthy microbiome composition among humans (and mice), typical microbiomes are enriched in the bacteria phyla *Firmicutes* and *Bacteroidetes*. Gut dysbiosis has been implicated in the pathogenesis of multiple diseases like inflammatory bowel diseases (IBD), autoimmune disorders (i.e. arthritis), diabetes, etc, while germ-free mice are protected in these models (Willits et al., 2021). Restoration of the “healthy” microbiome often corresponds to improvement in other health conditions. For example, obese rodents and patients have a significant increase in *Firmicutes* to the detriment of *Bacteroidetes*, (Carding et al., 2015), while a calorie-restricted diet that rescues gut dysbiosis also promotes weight loss (Carding et al., 2015). Interestingly, stroke and traumatic brain injury produce microbial colonization shifts towards increased gram-negative, pathogenic bacteria and reduced beneficial species like *Firmicutes* (Magne et al., 2020, Jogia and Ruitenberg, 2020). While the mechanisms by which CNS injury could drive specific shifts in gut microbial colonization remain unclear, these noted changes suggest that targeted investigation of host-microbiome response dynamics after traumatic SCI as a novel therapeutic target for the prevention of NB.

Few clinical studies have reported human microbiome shifts after SCI (Nicholson et al., 2019, Gungor et al., 2016, Zhang et al., 2019), but these studies tend to be underpowered and primarily associative in nature, making it difficult to determine whether microbiome changes play a causative role in outcomes after injury. Microbiome manipulation, through fecal material transplant or probiotic treatment, can improve locomotor outcomes in animal models of SCI (Zhang et al., 2018, Kigerl et al., 2016), however we are the first to identify and propose a role for a specific bacteria in the etiology of chronic abdominal pain and dysfunction after SCI. Specifically, *Anaeroplasma*, a gram-negative bacteria characterized as pathogenic (Jing et al., 2021), was increased in chronic SCI and is significantly increased in human patients with inflammation-driven colorectal cancer (Chen et al., 2021), irritable bowel syndrome presenting with constipation (Gates et al., 2023), and Crohn’s disease (Choo et al., 2022) highlighting the potential involvement of this microbe in diseases with overlapping symptom profiles to NB.

SCI patients are a greater risk of mortality from systemic infection and septicemia (Willing et al., 2010). The most common microbial mediator of sepsis is lipopolysaccharide (LPS) a membrane protein found on gram-negative bacetria (Weiterer et al., 2019) including Anaeroplasma. LPS can directly stimulate sensory neurons via Toll-like Receptor 4 (TL4) (Opal, 2010) inducing depolarization, initiating cell signaling, and driving gene expression changes critical for increased neuronal activity (Ochoa-Cortes et al., 2010). In addition to the direct effects of bacteria, short chain fatty acids (SCFAs) produced by microbes are used by gut wall epithelial cells as fuel for cellular metabolism (Willits et al., 2021). When there are shifts in microbial colonization, this can change SCFA abundance and negatively affect host cells through a variety of mechanisms. For example, butyrate can inhibit histone deacetylases (HDACs) (Barajon et al., 2009, Davie, 2003) responsible for regulating chromatin structure and, thereby, indirectly regulating host gene expression.

Increased transcript abundance of *lipopolysaccharide binding protein (lpb), peptidoglycan recognition protein 1 (pglyrp1), cathelicidin antimicrobial peptide (camp)* and other genes primarily involved in host defense response and interaction with biotic stimuli suggests the potential for microbiome-driven reprogramming of host-gene expression in primary afferents after SCI. In fact, colon-specific vagal afferents were hyperresponsive to autologous gut lumen contents 28 days after SCI, suggesting these sensory neurons may serve as a cellular substrate for gut bacteria to drive chronic abdominal pain signals and bypassing the damaged ascending tracts in the spinal cord. Cultured vagal afferents from naïve mice were hyperresponsive to stool from day 28 SCI mice, indicating the altered composition of the microbiome is sufficient to increase neuronal activation even in normal healthy cells. Taken together with recent work from Pradhananga et al. indicating cysteine proteases derived from commensal gut microbes, as well as LPS, increase electrical excitability of cultured vagal afferents (Hou and Wang, 2001), these data point to a process by which gut dysbiosis could sensitize enteric neurons and vagal afferents to amplify pain transmission to the brain, bypassing spinal processing, as a mechanism for the perceptual experience of abdominal pain in SCI patients.

### 4.3 Limitations and future directions

We acknowledge the importance of sex as a biological variable, however bladder voiding efficacy following SCI is low in males, leading to reduced survival rate and presenting a confounding source of visceral injury and/or pain separate from the intended effects of SCI. For this reason and to ensure proper sample sizes, our study is restricted to females only. While it is possible that CGRP may have a limited impact in male rodents (Waldecker et al., 2008), monoclonal antibodies targeting CGRP to treat migraine have been shown equally safe and effective in males and females (Paige et al., 2022, Ornello et al., 2021). In addition, we do not directly test the cause-and-effect relationship of CGRP on NB-like phenotypes in our mouse model, but this represents only one of the novel hypotheses generated as a result of our comprehensive phenotyping of NB in this mouse model of SCI. Previous literature suggests that antidromic activity results in peripheral CGRP release and our current data implicate CGRP in subsequent organ dysfunction, but it is possible that the increased CGRP occurs downstream of alterations to colon structure and function. We successfully achieved our primary objective for this study which was to characterize NB-like phenotypic, transcriptomic, and neuronal changes after SCI to generate testable hypotheses regarding the mechanisms underlying NB. This work provides critical validation of the SCI mouse model for the study of NB AND provides the foundation for subsequent hypothesis testing on the role of antidromic activity, CGRP signaling, and gut dysbiosis on the development and chronicity of NB.

## 5 Conclusions

The current findings begin to address the critical knowledge gap surrounding SCI-induced NB by identifying the cellular and molecular processes engaged in visceral tissues following SCI. NB significantly affects the quality of life for people living with SCI, however therapeutic options are limited in part due to a lack of understanding of NB pathogenesis. Continued investigation into the mechanisms involved in initiating and maintaining bowel dysfunction after SCI is critically important to the development of evidence-based, novel therapeutic options for bowel dysfunction and pain after SCI.

## CRediT authorship contribution statement

**Adam B. Willits:** Writing – review & editing, Writing – original draft, Visualization, Validation, Investigation, Formal analysis, Data curation, Conceptualization. **Leena Kader:** Writing – review & editing, Visualization, Investigation, Formal analysis. **Olivia Eller:** Visualization, Investigation, Formal analysis. **Emily Roberts:** Visualization, Investigation, Formal analysis. **Bailey Bye:** Visualization, Investigation, Formal analysis. **Taylor Strope:** Visualization, Investigation, Formal analysis. **Bret D. Freudenthal:** Writing – review & editing, Methodology, Conceptualization. **Shahid Umar:** Supervision, Resources, Methodology, Formal analysis, Conceptualization. **Sree Chintapalli:** Writing – review & editing, Visualization, Supervision, Resources, Methodology, Formal analysis, Conceptualization. **Kartik Shankar:** Writing – review & editing, Visualization, Software, Resources, Methodology, Investigation, Formal analysis. **Dong Pei:** Writing – review & editing, Visualization, Software, Resources, Methodology, Formal analysis. **Julie Christianson:** Writing – review & editing, Supervision, Resources, Methodology, Investigation, Formal analysis. **Kyle M. Baumbauer:** Writing – review & editing, Validation, Supervision, Resources, Methodology, Investigation, Funding acquisition, Formal analysis, Conceptualization. **Erin E. Young:** Writing – review & editing, Writing – original draft, Visualization, Validation, Supervision, Software, Resources, Methodology, Investigation, Funding acquisition, Formal analysis, Conceptualization.

## Declaration of competing interest

The authors declare that they have no known competing financial interests or personal relationships that could have appeared to influence the work reported in this paper.

## Data Availability

Data will be made available on request.

## References

[b0005] Ahmed I., Roy B.C., Raach R.T., Owens S.M., Xia L., Anant S., Sampath V., Umar S. (2018). Enteric infection coupled with chronic Notch pathway inhibition alters colonic mucus composition leading to dysbiosis, barrier disruption and colitis. PLoS One.

[b0010] Anderson K.D. (2004). Targeting recovery: priorities of the spinal cord-injured population. J. Neurotrauma.

[b0015] Barajon I., Serrao G., Arnaboldi F., Opizzi E., Ripamonti G., Balsari A., Rumio C. (2009). Toll-like receptors 3, 4, and 7 are expressed in the enteric nervous system and dorsal root ganglia. J. Histochem. Cytochem..

[b0020] Bardou P., Mariette J., Escudie F., Djemiel C., Klopp C. (2014). jvenn: an interactive Venn diagram viewer. BMC Bioinf..

[b0025] Basso D.M., Fisher L.C., Anderson A.J., Jakeman L.B., McTigue D.M., Popovich P.G. (2006). Basso Mouse Scale for locomotion detects differences in recovery after spinal cord injury in five common mouse strains. J. Neurotrauma.

[b0030] Bedi S.S., Yang Q., Crook R.J., Du J., Wu Z., Fishman H.M., Grill R.J., Carlton S.M., Walters E.T. (2010). Chronic spontaneous activity generated in the somata of primary nociceptors is associated with pain-related behavior after spinal cord injury. J. Neurosci..

[b0035] Bhalala O.G., Pan L., North H., McGuire T., Kessler J.A. (2013). Generation of Mouse Spinal Cord Injury. Bio Protoc.

[b0040] Boland J.W., Boland E.G. (2017). Pharmacological therapies for opioid induced constipation in adults with cancer. BMJ.

[b0045] Brink L.R., Matazel K., Piccolo B.D., Bowlin A.K., Chintapalli S.V., Shankar K., Yeruva L. (2019). Neonatal Diet Impacts Bioregional Microbiota Composition in Piglets Fed Human Breast Milk or Infant Formula. J. Nutr..

[b0050] Brink L.R., Mercer K.E., Piccolo B.D., Chintapalli S.V., Elolimy A., Bowlin A.K., Matazel K.S., Pack L., Adams S.H., Shankar K. (2020). Neonatal diet alters fecal microbiota and metabolome profiles at different ages in infants fed breast milk or formula. Am. J. Clin. Nutr..

[b0055] Buettner M., Lochner M. (2016). Development and Function of Secondary and Tertiary Lymphoid Organs in the Small Intestine and the Colon. Front. Immunol..

[b0060] Burke D., Fullen B.M., Stokes D., Lennon O. (2017). Neuropathic pain prevalence following spinal cord injury: A systematic review and meta-analysis. Eur. J. Pain.

[b0065] Burke D., Fullen B.M., Lennon O. (2019). Pain profiles in a community dwelling population following spinal cord injury: a national survey. J. Spinal Cord Med..

[b0070] Carding S., Verbeke K., Vipond D.T., Corfe B.M., Owen L.J. (2015). Dysbiosis of the gut microbiota in disease. Microb. Ecol. Health Dis..

[b0075] Carlton S.M., Du J., Tan H.Y., Nesic O., Hargett G.L., Bopp A.C., Yamani A., Lin Q., Willis W.D., Hulsebosch C.E. (2009). Peripheral and central sensitization in remote spinal cord regions contribute to central neuropathic pain after spinal cord injury. Pain.

[b0080] Center, N.S.C.I.S. (2019). Facts and Figures at a Glance. In U.o. Birmingham, ed.

[b0085] Chen Q., He Z., Zhuo Y., Li S., Yang W., Hu L., Zhong H. (2021). Rubidium chloride modulated the fecal microbiota community in mice. BMC Microbiol..

[b0090] Cheriyan T., Ryan D.J., Weinreb J.H., Cheriyan J., Paul J.C., Lafage V., Kirsch T., Errico T.J. (2014). Spinal cord injury models: a review. Spinal Cord.

[b0095] Choo C., Mahurkar-Joshi S., Dong T.S., Lenhart A., Lagishetty V., Jacobs J.P., Labus J.S., Jaffe N., Mayer E.A., Chang L. (2022). Colonic mucosal microbiota is associated with bowel habit subtype and abdominal pain in patients with irritable bowel syndrome. Am. J. Physiol. Gastrointest. Liver Physiol..

[b0100] Christianson, J.A., Bielefeldt, K., Malin, S.A., and Davis, B.M. (2010). Neonatal colon insult alters growth factor expression and TRPA1 responses in adult mice. Pain *151*, 540-549. S0304-3959(10)00512-9 [pii] 10.1016/j.pain.2010.08.029 [doi]. 10.1016/j.pain.2010.08.029 PMC2955795 20850221

[b0105] Cruz-Almeida Y., Martinez-Arizala A., Widerstrom-Noga E.G. (2005). Chronicity of pain associated with spinal cord injury: A longitudinal analysis. J. Rehabil. Res. Dev..

[b0110] Davie J.R. (2003). Inhibition of histone deacetylase activity by butyrate. J. Nutr..

[b0115] Eller O.C., Stair R.N., Neal C., Rowe P.S.N., Nelson-Brantley J., Young E.E., Baumbauer K.M. (2022). Comprehensive phenotyping of cutaneous afferents reveals early-onset alterations in nociceptor response properties, release of CGRP, and hindpaw edema following spinal cord injury. Neurobiol Pain.

[b0120] Emmanuel A. (2019). Neurogenic bowel dysfunction. F1000Res.

[b0125] Faaborg P.M., Finnerup N.B., Christensen P., Krogh K. (2013). Abdominal Pain: A Comparison between Neurogenic Bowel Dysfunction and Chronic Idiopathic Constipation. Gastroenterol. Res. Pract..

[b0130] Finnerup N.B., Faaborg P., Krogh K., Jensen T.S. (2008). Abdominal pain in long-term spinal cord injury. Spinal Cord.

[b0135] Fu H., Zhang T., Huang R., Yang Z., Liu C., Li M., Fang F., Xu F. (2017). Calcitonin gene-related peptide protects type II alveolar epithelial cells from hyperoxia-induced DNA damage and cell death. Exp. Ther. Med..

[b0140] Gale J.R., Gedeon J.Y., Donnelly C.J., Gold M.S. (2022). Local translation in primary afferents and its contribution to pain. Pain.

[b0145] Gates T.J., Yuan C., Shetty M., Kaiser T., Nelson A.C., Chauhan A., Starr T.K., Staley C., Subramanian S. (2023). Fecal Microbiota Restoration Modulates the Microbiome in Inflammation-Driven Colorectal Cancer. Cancers (basel).

[b0150] Grunkemeier, D.M., Cassara, J.E., Dalton, C.B., and Drossman, D.A. (2007). The narcotic bowel syndrome: clinical features, pathophysiology, and management. Clin Gastroenterol Hepatol *5*, 1126-1139; quiz 1121-1122. 10.1016/j.cgh.2007.06.013. 10.1016/j.cgh.2007.06.013 PMC2074872 17916540

[b0155] Gungor B., Adiguzel E., Gursel I., Yilmaz B., Gursel M. (2016). Intestinal Microbiota in Patients with Spinal Cord Injury. PLoS One.

[b0160] Haring M., Fatt M., Kupari J. (2020). Protocol to Prepare Single-Cell Suspensions from Mouse Vagal Sensory Ganglia for Transcriptomic Studies. STAR Protoc.

[b0165] Hou L., Wang X. (2001). PKC and PKA, but not PKG mediate LPS-induced CGRP release and [Ca(2+)](i) elevation in DRG neurons of neonatal rats. J. Neurosci. Res..

[b0170] Hubscher C.H., Herrity A.N., Williams C.S., Montgomery L.R., Willhite A.M., Angeli C.A., Harkema S.J. (2018). Improvements in bladder, bowel and sexual outcomes following task-specific locomotor training in human spinal cord injury. PLoS One.

[b0175] Hulsebosch C.E., Hains B.C., Crown E.D., Carlton S.M. (2009). Mechanisms of chronic central neuropathic pain after spinal cord injury. Brain Res. Rev..

[b0180] Iyengar S., Ossipov M.H., Johnson K.W. (2017). The role of calcitonin gene-related peptide in peripheral and central pain mechanisms including migraine. Pain.

[b0185] Jing Y., Yu Y., Bai F., Wang L., Yang D., Zhang C., Qin C., Yang M., Zhang D., Zhu Y. (2021). Effect of fecal microbiota transplantation on neurological restoration in a spinal cord injury mouse model: involvement of brain-gut axis. Microbiome.

[b0190] Jogia T., Ruitenberg M.J. (2020). Traumatic Spinal Cord Injury and the Gut Microbiota: Current Insights and Future Challenges. Front. Immunol..

[b0195] Johansson M.E., Phillipson M., Petersson J., Velcich A., Holm L., Hansson G.C. (2008). The inner of the two Muc2 mucin-dependent mucus layers in colon is devoid of bacteria. PNAS.

[b0200] Johns, J.S., Krogh, K., Ethans, K., Chi, J., Queree, M., Eng, J.J., and Spinal Cord Injury Research Evidence, T. (2021). Pharmacological Management of Neurogenic Bowel Dysfunction after Spinal Cord Injury and Multiple Sclerosis: A Systematic Review and Clinical Implications. J. Clin. Med. 10. 10.3390/jcm10040882. 10.3390/jcm10040882 PMC7926827 33671492

[b0205] Kamp E.H., Jones R.C., Tillman S.R., Gebhart G.F. (2003). Quantitative assessment and characterization of visceral nociception and hyperalgesia in mice. Am. J. Physiol. Gastrointest. Liver Physiol..

[b0210] Kee Z., Kodji X., Brain S.D. (2018). The Role of Calcitonin Gene Related Peptide (CGRP) in Neurogenic Vasodilation and Its Cardioprotective Effects. Front. Physiol..

[b0215] Kigerl K.A., Lai W., Rivest S., Hart R.P., Satoskar A.R., Popovich P.G. (2007). Toll-like receptor (TLR)-2 and TLR-4 regulate inflammation, gliosis, and myelin sparing after spinal cord injury. J. Neurochem..

[b0220] Kigerl K.A., Hall J.C., Wang L., Mo X., Yu Z., Popovich P.G. (2016). Gut dysbiosis impairs recovery after spinal cord injury. J. Exp. Med..

[b0225] Kress M., Guthmann C., Averbeck B., Reeh P.W. (1999). Calcitonin gene-related peptide and prostaglandin E2 but not substance P release induced by antidromic nerve stimulation from rat skin in vitro. Neuroscience.

[b0230] Krogh K., Nielsen J., Djurhuus J.C., Mosdal C., Sabroe S., Laurberg S. (1997). Colorectal function in patients with spinal cord lesions. Dis. Colon Rectum.

[b0235] Krogh K., Mosdal C., Laurberg S. (2000). Gastrointestinal and segmental colonic transit times in patients with acute and chronic spinal cord lesions. Spinal Cord.

[b0240] Kumaravel T.S., Vilhar B., Faux S.P., Jha A.N. (2009). Comet Assay measurements: a perspective. Cell Biol. Toxicol..

[b0245] Levi R., Hultling C., Nash M.S., Seiger A. (1995). The Stockholm spinal cord injury study: 1. Medical problems in a regional SCI population. Paraplegia.

[b0250] Liao Y., Wang J., Jaehnig E.J., Shi Z., Zhang B. (2019). WebGestalt 2019: gene set analysis toolkit with revamped UIs and APIs. Nucleic Acids Res..

[b0255] Magne F., Gotteland M., Gauthier L., Zazueta A., Pesoa S., Navarrete P., Balamurugan R. (2020). The Firmicutes/Bacteroidetes Ratio: A Relevant Marker of Gut Dysbiosis in Obese Patients?. Nutrients.

[b0260] Malin S.A., Davis B.M., Molliver D.C. (2007). Production of dissociated sensory neuron cultures and considerations for their use in studying neuronal function and plasticity. Nat. Protoc..

[b0265] Malin S., Molliver D., Christianson J.A., Schwartz E.S., Cornuet P., Albers K.M., Davis B.M. (2011). TRPV1 and TRPA1 function and modulation are target tissue dependent. J. Neurosci..

[b0270] McRoberts J.A., Coutinho S.V., Marvizon J.C., Grady E.F., Tognetto M., Sengupta J.N., Ennes H.S., Chaban V.V., Amadesi S., Creminon C. (2001). Role of peripheral N-methyl-D-aspartate (NMDA) receptors in visceral nociception in rats. Gastroenterology.

[b0275] Mercer K.E., Yeruva L., Pack L., Graham J.L., Stanhope K.L., Chintapalli S.V., Wankhade U.D., Shankar K., Havel P.J., Adams S.H., Piccolo B.D. (2020). Xenometabolite signatures in the UC Davis type 2 diabetes mellitus rat model revealed using a metabolomics platform enriched with microbe-derived metabolites. Am. J. Physiol. Gastrointest. Liver Physiol..

[b0280] Minerbi A., Gonzalez E., Brereton N.J.B., Anjarkouchian A., Dewar K., Fitzcharles M.A., Chevalier S., Shir Y. (2019). Altered microbiome composition in individuals with fibromyalgia. Pain.

[b0285] Monteiro S., Salgado A.J., Silva N.A. (2018). Immunomodulation as a neuroprotective strategy after spinal cord injury. Neural Regen. Res..

[b0290] Nicholson S.E., Watts L.T., Burmeister D.M., Merrill D., Scroggins S., Zou Y., Lai Z., Grandhi R., Lewis A.M., Newton L.M. (2019). Moderate Traumatic Brain Injury Alters the Gastrointestinal Microbiome in a Time-Dependent Manner. Shock.

[b0295] Nielsen S.D., Faaborg P.M., Christensen P., Krogh K., Finnerup N.B. (2017). Chronic abdominal pain in long-term spinal cord injury: a follow-up study. Spinal Cord.

[b0300] Ochoa-Cortes F., Ramos-Lomas T., Miranda-Morales M., Spreadbury I., Ibeakanma C., Barajas-Lopez C., Vanner S. (2010). Bacterial cell products signal to mouse colonic nociceptive dorsal root ganglia neurons. Am. J. Physiol. Gastrointest. Liver Physiol..

[b0305] Odem M.A., Bavencoffe A.G., Cassidy R.M., Lopez E.R., Tian J., Dessauer C.W., Walters E.T. (2018). Isolated nociceptors reveal multiple specializations for generating irregular ongoing activity associated with ongoing pain. Pain.

[b0310] Odem M.A., Lacagnina M.J., Katzen S.L., Li J., Spence E.A., Grace P.M., Walters E.T. (2019). Sham surgeries for central and peripheral neural injuries persistently enhance pain-avoidance behavior as revealed by an operant conflict test. Pain.

[b0315] Opal S.M. (2010). Endotoxins and other sepsis triggers. Contrib. Nephrol..

[b0320] Ornello R., Baraldi C., Guerzoni S., Lambru G., Fuccaro M., Raffaelli B., Gendolla A., Barbanti P., Aurilia C., Cevoli S. (2021). Gender Differences in 3-Month Outcomes of Erenumab Treatment-Study on Efficacy and Safety of Treatment With Erenumab in Men. Front. Neurol..

[b0325] Paige C., Plasencia-Fernandez I., Kume M., Papalampropoulou-Tsiridou M., Lorenzo L.E., David E.T., He L., Mejia G.L., Driskill C., Ferrini F. (2022). A Female-Specific Role for Calcitonin Gene-Related Peptide (CGRP) in Rodent Pain Models. J. Neurosci..

[b0330] Peirs C., Seal R.P. (2016). Neural circuits for pain: Recent advances and current views. Science.

[b0335] Pierce A.N., Zhang Z., Fuentes I.M., Wang R., Ryals J.M., Christianson J.A. (2015). Neonatal vaginal irritation results in long-term visceral and somatic hypersensitivity and increased hypothalamic-pituitary-adrenal axis output in female mice. Pain.

[b0340] Pradhananga S., Tashtush A.A., Allen-Vercoe E., Petrof E.O., Lomax A.E. (2020). Protease-dependent excitation of nodose ganglion neurons by commensal gut bacteria. J. Physiol..

[b0345] Rekand T., Hagen E.M., Gronning M. (2012). Chronic pain following spinal cord injury. Tidsskr. Nor. Laegeforen..

[b0350] Round A.M., Joo M.C., Barakso C.M., Fallah N., Noonan V.K., Krassioukov A.V. (2021). Neurogenic Bowel in Acute Rehabilitation Following Spinal Cord Injury: Impact of Laxatives and Opioids. J. Clin. Med..

[b0355] Russell F.A., King R., Smillie S.J., Kodji X., Brain S.D. (2014). Calcitonin gene-related peptide: physiology and pathophysiology. Physiol. Rev..

[b0360] Rust R., Kaiser J. (2017). Insights into the Dual Role of Inflammation after Spinal Cord Injury. J. Neurosci..

[b0365] Schindelin J., Arganda-Carreras I., Frise E., Kaynig V., Longair M., Pietzsch T., Preibisch S., Rueden C., Saalfeld S., Schmid B. (2012). Fiji: an open-source platform for biological-image analysis. Nat. Methods.

[b0370] Schumacher T.N., Thommen D.S. (2022). Tertiary lymphoid structures in cancer. Science.

[b0375] Siddall P.J., McClelland J.M., Rutkowski S.B., Cousins M.J. (2003). A longitudinal study of the prevalence and characteristics of pain in the first 5 years following spinal cord injury. Pain.

[b0380] Sluka K.A. (1996). Pain mechanisms involved in musculoskeletal disorders. J. Orthop. Sports Phys. Ther..

[b0385] Sorkin L.S., Eddinger K.A., Woller S.A., Yaksh T.L. (2018). Origins of antidromic activity in sensory afferent fibers and neurogenic inflammation. Semin. Immunopathol..

[b0390] Stone J.M., Nino-Murcia M., Wolfe V.A., Perkash I. (1990). Chronic gastrointestinal problems in spinal cord injury patients: a prospective analysis. Am. J. Gastroenterol..

[b0395] Sun X., Jones Z.B., Chen X.M., Zhou L., So K.F., Ren Y. (2016). Multiple organ dysfunction and systemic inflammation after spinal cord injury: a complex relationship. J. Neuroinflammation.

[b0400] Team, S.C. (2023). What's the Real Spinal Cord Injury Cost in 2023? https://www.spinalcord.com/blog/what-is-the-real-spinal-cord-injury-cost.

[b0405] Toth C.C., Willis D., Twiss J.L., Walsh S., Martinez J.A., Liu W.Q., Midha R., Zochodne D.W. (2009). Locally synthesized calcitonin gene-related Peptide has a critical role in peripheral nerve regeneration. J. Neuropathol. Exp. Neurol..

[b0410] Vance K.M., Rogers R.C., Hermann G.E. (2015). NMDA receptors control vagal afferent excitability in the nucleus of the solitary tract. Brain Res..

[b0415] Vanuytsel T., Tack J.F., Boeckxstaens G.E. (2014). Treatment of abdominal pain in irritable bowel syndrome. J. Gastroenterol..

[b0420] Vilz T.O., Overhaus M., Stoffels B., Websky M., Kalff J.C., Wehner S. (2012). Functional assessment of intestinal motility and gut wall inflammation in rodents: analyses in a standardized model of intestinal manipulation. J. Vis. Exp..

[b0425] Waldecker M., Kautenburger T., Daumann H., Busch C., Schrenk D. (2008). Inhibition of histone-deacetylase activity by short-chain fatty acids and some polyphenol metabolites formed in the colon. J. Nutr. Biochem..

[b0430] Walters E.T. (2012). Nociceptors as chronic drivers of pain and hyperreflexia after spinal cord injury: an adaptive-maladaptive hyperfunctional state hypothesis. Front. Physiol..

[b0435] Wankhade U.D., Zhong Y., Lazarenko O.P., Chintapalli S.V., Piccolo B.D., Chen J.R., Shankar K. (2019). Sex-Specific Changes in Gut Microbiome Composition following Blueberry Consumption in C57BL/6J Mice. Nutrients.

[b0440] Weiterer S., Frick S., Lichtenstern C., Hug A., Uhle F., Weigand M.A., Hundt G., Siegler B.H. (2019). Sepsis in mechanically ventilated patients with spinal cord injury: a retrospective analysis. Spinal Cord.

[b0445] Willing B.P., Dicksved J., Halfvarson J., Andersson A.F., Lucio M., Zheng Z., Jarnerot G., Tysk C., Jansson J.K., Engstrand L. (2010). A pyrosequencing study in twins shows that gastrointestinal microbial profiles vary with inflammatory bowel disease phenotypes. Gastroenterology.

[b0450] Willits A.B., Grossi V., Glidden N.C., Hyams J.S., Young E.E. (2021). Identification of a Pain-Specific Gene Expression Profile for Pediatric Recurrent Abdominal Pain. Front Pain Res (lausanne).

[b0455] Yang Q., Wu Z., Hadden J.K., Odem M.A., Zuo Y., Crook R.J., Frost J.A., Walters E.T. (2014). Persistent pain after spinal cord injury is maintained by primary afferent activity. J. Neurosci..

[b0460] Yip J.L.K., Balasuriya G.K., Spencer S.J., Hill-Yardin E.L. (2022). Examining enteric nervous system function in rat and mouse: an interspecies comparison of colonic motility. Am. J. Physiol. Gastrointest. Liver Physiol..

[b0465] Zhang Y., Hu W. (2013). Mouse enteric neuronal cell culture. Methods Mol. Biol..

[b0470] Zhang C., Zhang W., Zhang J., Jing Y., Yang M., Du L., Gao F., Gong H., Chen L., Li J. (2018). Gut microbiota dysbiosis in male patients with chronic traumatic complete spinal cord injury. J. Transl. Med..

[b0475] Zhang C., Jing Y., Zhang W., Zhang J., Yang M., Du L., Jia Y., Chen L., Gong H., Li J. (2019). Dysbiosis of gut microbiota is associated with serum lipid profiles in male patients with chronic traumatic cervical spinal cord injury. Am. J. Transl. Res..

[b0480] Zhou Y., Zhou B., Pache L., Chang M., Khodabakhshi A.H., Tanaseichuk O., Benner C., Chanda S.K. (2019). Metascape provides a biologist-oriented resource for the analysis of systems-level datasets. Nat. Commun..

